# Intratumoral co‐injection of NK cells and NKG2A‐neutralizing monoclonal antibodies

**DOI:** 10.15252/emmm.202317804

**Published:** 2023-10-02

**Authors:** Ignacio Melero, Maria C Ochoa, Carmen Molina, Sandra Sanchez‐Gregorio, Saray Garasa, Carlos Luri‐Rey, Sandra Hervas‐Stubbs, Noelia Casares, Edurne Elizalde, Gabriel Gomis, Assunta Cirella, Pedro Berraondo, Alvaro Teijeira, Maite Alvarez

**Affiliations:** ^1^ Program for Immunology and Immunotherapy, CIMA Universidad de Navarra Pamplona Spain; ^2^ Navarra Institute for Health Research (IdiSNA) Pamplona Spain; ^3^ Centro de Investigación Biomédica en Red de Cáncer (CIBERONC) Madrid Spain; ^4^ Departments of Immunology and Oncology Clínica Universidad de Navarra Pamplona Spain; ^5^ Cell Therapy, Stem Cells and Tissue Group Biocruces Bizkaia Health Research Institute Barakaldo Spain; ^6^ Research Unit, Basque Center for Blood Transfusion and Human Tissues Osakidetza Galdakao Spain

**Keywords:** HLA‐E, intratumoral immunotherapy, NK, NKG2A, Qa‐1^b^, Cancer, Immunology

## Abstract

NK‐cell reactivity against cancer is conceivably suppressed in the tumor microenvironment by the interaction of the inhibitory receptor NKG2A with the non‐classical MHC‐I molecules HLA‐E in humans or Qa‐1^b^ in mice. We found that intratumoral delivery of NK cells attains significant therapeutic effects only if co‐injected with anti‐NKG2A and anti‐Qa‐1^b^ blocking monoclonal antibodies against solid mouse tumor models. Such therapeutic activity was contingent on endogenous CD8 T cells and type‐1 conventional dendritic cells (cDC1). Moreover, the anti‐tumor effects were enhanced upon combination with systemic anti‐PD‐1 mAb treatment and achieved partial abscopal efficacy against distant non‐injected tumors. In xenografted mice bearing HLA‐E‐expressing human cancer cells, intratumoral co‐injection of activated allogeneic human NK cells and clinical‐grade anti‐NKG2A mAb (monalizumab) synergistically achieved therapeutic effects. In conclusion, these studies provide evidence for the clinical potential of intratumoral NK cell‐based immunotherapies that exert their anti‐tumor efficacy as a result of eliciting endogenous T‐cell responses.

## Graphical abstract

An approach that involves intratumoral injections of NK cells together with neutralizing antibodies against the NKG2A inhibitory receptor was developed, aiming at extending the efficacy of NK cell‐based immunotherapies to solid malignances.

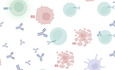

**The paper explaned**

**Problem**

The efficacy of NK cell‐based therapies has been very disappointing against solid tumors due to limited NK cell traffic into the cancer tissue microenvironment. In addition, the expression on the tumor‐cell surface of molecules recognized by NK‐inhibitory receptors accounts for the poor efficiency of transferred NK cells at clearing tumors. In the absence of ensuing adaptive immunity, short NK‐cell persistence also precludes efficacy.

**Results**

The intratumoral co‐injections of pre‐activated NK cells together with NKG2A neutralizing antibodies tampering with this inhibitory receptor achieves anti‐tumoral synergy in mouse tumor models in a manner dependent on CD8 T cells and cDC1 cells.

**Impact**

GMP‐cultured NK cells and clinical‐grade antibodies that block NKG2A (i.e. monalizumab) are available, and, accordingly, an immunotherapeutic strategy based on intratumoral delivery of NK cells and anti‐NKG2A mAbs is clinically feasible, as shown here in tumor‐xenografted mice intratumorally treated with human NK cells and monalizumab.

## Introduction

NK cells are innate lymphocytes able to spontaneously eliminate cancer and virus infected cells in an MHC class‐I unrestricted manner (Wolf *et al*, 2023). There is strong evidence for the importance of NK cells in the control of tumors (O'Sullivan *et al*, 2012; Wolf *et al*, 2023) and thus, NK cell‐based immunotherapies have been extensively explored in many cancer types (Lamers‐Kok *et al*, 2022). Unfortunately, the clinical benefit of NK cell‐based therapies has been, for the most part, rather disappointing, since objective response and overall survival improvements have been very limited in the clinic (Lamers‐Kok *et al*, 2022). An important caveat is that contrary to T cells, NK cells do not experience persistence as a result of clonal expansions. In addition, NK cells are prey to numerous immunosuppressive mechanisms in the tumor microenvironment (TME). Indeed, they are susceptible to the immunosuppressive activity of regulatory T cells (Tregs), myeloid‐derived suppressor cells (MDSC), immunosuppressive cytokines, such as TGFβ and IL‐10, and hypoxia (Myers & Miller, 2021; Dunai *et al*, 2022; Zhang *et al*, 2022).

The expansion of NK cells in culture from peripheral blood is relatively simple (Myers & Miller, 2021; Lamers‐Kok *et al*, 2022). The most successful GMP‐compliant approaches involve the use of recombinant interleukin‐2 (IL‐2) and IL‐15, as well as the 4‐1BB/4‐1BBL costimulation pathway, to grow large numbers of NK cells that can be infused into cancer patients (Phan *et al*, 2016). The approach offers promising results in the treatment of myeloid hematological malignancies including acute myeloid leukemia (AML) (Miller *et al*, 2005; Locatelli *et al*, 2014). In solid tumors, experience is limited and chiefly involves cases of neuroblastoma (Modak *et al*, 2018). Previously, attempts to infuse cytokine‐activated T and NK cells as adoptive cell therapies have shown tolerability, but have provided limited evidence for clinical benefit (Lamers‐Kok *et al*, 2022; Wolf *et al*, 2023).

To make the most of NK cells in cancer therapy, their activating mechanisms upon target engagement can be exploited. For instance, using antibody‐dependent cellular cytotoxicity (ADCC) mediated by CD16a recognition of antibody‐coated target cells (Pinto *et al*, 2022). Currently, agonist bispecific antibodies engaging activating receptors on NK cells such as NKp46 and NKG2D are under development (Pinto *et al*, 2022; Gauthier *et al*, 2023). A notable advance has come from retroviral engineering of cultured NK cells to express anti‐CD19 chimeric antigen receptors (CAR) signaling via CD3ζ and 4‐1BB. Such CAR‐NK cells have shown therapeutic activity against acute lymphoblastic leukemia (ALL) (Liu *et al*, 2020) and have the advantage of being of allogeneic origin without the need for their expansion from an ill patient given the fact they can be available “off‐the‐shelf” once frozen (Lamers‐Kok *et al*, 2022).

NK cells may directly destroy malignant cells, but indirectly they can also facilitate ensuing adaptive T‐cell immunity against cancer as a result of cytokine production and by supplying antigens from killed cells for cross‐priming by dendritic cells to CD8 T cells (Pitt *et al*, 2017; Barry *et al*, 2018; Böttcher *et al*, 2018; Galluzzi *et al*, 2020, 2023; Minute *et al*, 2020).

NK cells are tightly regulated through an actionable delicate balance of activating and inhibitory surface receptors (Muntasell *et al*, 2017) that is often disrupted during prolonged stimulation, such as chronic viral infections or exposure to tumors that reportedly result in NK‐cell exhaustion and dysfunction (Alvarez *et al*, 2019). One of the checkpoint inhibitors responsible for such regulation is the dimeric inhibitory receptor NKG2A/CD94 (Lee *et al*, 1998b), which is also expressed on some CD8 T cells (Borst *et al*, 2020). NKG2A expression levels are highly upregulated during activation (Alvarez *et al*, 2019). Inhibition is elicited when NKG2A/CD94 binds to the non‐classical MHC class‐I (MHC‐I) HLA‐E in humans (Lee *et al*, 1998b) or its homologous protein in mice, Qa‐1^b^ (Vance *et al*, 1999), on the surface of target cells. This ligand‐receptor interaction causes the recruitment of the protein tyrosine phosphatase SHP‐1 to the cytotoxic signaling synapse and thereby inhibits activating receptors (van Hall *et al*, 2019). Interestingly, NKG2A shares its ligand with the activating receptor NKG2C that also heterodimerizes with CD94 (Braud *et al*, 1998). The NKG2C^+^ population as compared to the NKG2A^+^ subset is more cytolytic and expanded in humans pre‐exposed to cytomegalovirus (Gumá *et al*, 2004; López‐Botet *et al*, 2023). NKG2A affinity for HLA‐E has been shown to be at least six‐fold higher than the affinity measured for the activating NKG2C receptor (Kaiser *et al*, 2005) and therefore in a scenario of NKG2A expression, cytotoxicity suppression by its ligands conceivably prevails.

In cancer, HLA‐E expression has been correlated with poor prognosis in patients with glioblastoma, pancreatic cancer and head and neck squamous cell carcinoma (HNSCC), among others (van Montfoort *et al*, 2018; Hiraoka *et al*, 2020; Wu *et al*, 2020), a fact that is indicative of the potential of the HLA‐E‐NKG2A pathway as a target for immunotherapeutic interventions (Andre *et al*, 2018). Indeed, systemic neutralization of NKG2A has been shown to unleash a strong anti‐tumor response in mouse preclinical studies by increasing NK and/or CD8 T‐cell effector functions, which were further enhanced when combined with anti‐PD‐(L)1 therapy (Andre *et al*, 2018; van Montfoort *et al*, 2018). These studies highlighted the importance of the NKG2A inhibitory pathway for the function of both NK and CD8 T cells. These notions prompted the development of the first‐in‐class humanized IgG4 anti‐NKG2A antibody monalizumab (Andre *et al*, 2018) in various clinical trials, in which monalizumab is given intravenously, alone or in combination with cetuximab (anti‐EGFR), trastuzumab (anti‐HER2) or along with immune checkpoint inhibitors (ICI). Such treatment has, thus far, shown a tolerable safety profile in humans (Andre *et al*, 2018; Tinker *et al*, 2019; Borst *et al*, 2020; Galot *et al*, 2021; Herbst *et al*, 2022). It has been demonstrated that monalizumab's efficacy strongly depends on HLA‐E expression on tumor cells (Lee *et al*, 2022). However, when used as a monotherapy, monalizumab has not shown evidence for meaningful clinical activity that, by contrast, has been substantiated when combined with durvalumab (Herbst *et al*, 2022).

In this study, we evaluated the immunotherapeutic efficacy of activated NK cells given locally inside tumors, along with strategies that neutralize the NKG2A inhibitory pathway and thereby unleash robust local and systemic adaptive immune responses ultimately capable of better controlling tumor progression. NK cells were administered intratumorally in order to circumvent the limitations in terms of trafficking (Melero *et al*, 2014) and low bioavailability of the agents in the tumor tissue microenvironment. Intratumoral release of several agents (Melero *et al*, 2021), including immune cells (Cherkassky *et al*, 2022), has resulted in powerful immunotherapeutic effects not only against the injected malignant lesions but often also against metastases that had not been directly treated (Melero *et al*, 2021; Olivera *et al*, 2023). The results are reminiscent of various preclinical and clinical approaches to locally deliver adoptive T‐cell therapy (Cherkassky *et al*, 2022). Previous attempts to directly inject pre‐activated NK cells inside established mouse tumors exerted very modest therapeutic effects (Ishikawa *et al*, 2004; Capobianco *et al*, 2008).

Here, intratumoral delivery of NK cells along with the concomitant neutralization of the NKG2A inhibitory pathway led to strong and durable local and systemic anti‐tumor responses that were observed in syngeneic mouse tumor models and in immunodeficient mice engrafted with human HLA‐E^+^ tumors, which in the latter case were intratumorally treated with human NK cells.

## Results

### Neutralization of the NKG2A pathway during NK‐cell local adoptive therapy improves anti‐tumor activity

To neutralize the NKG2A receptor in mouse studies, we used a rat IgG2a mAb against NKG2A generated by Dr. Raulet's group (Vance *et al*, 1999), a mAb that can also bind to NKG2C and NKG2E. This is why we first evaluated the expression of NKG2A and NKG2C in activated NK cells. As a source of activated mouse NK cells, T and B‐cell deficient RAG1^−/−^ mice were pre‐treated with a hydrodynamic injection of an expression plasmid encoding a fusion protein of IL‐15 with apolipoprotein A as previously described (Ochoa *et al*, 2013; Alvarez *et al*, 2020a). The staining of spleen‐derived isolated NK cells showed that only NKG2A, but not NKG2C were expressed on the cell surface (Appendix Fig S1A and B). Therefore, the functional effect of the anti‐NKG2A/C/E mAb (20d5 clone) would be attributable solely to its binding to NKG2A. For simplicity, we will hereafter name this mAb anti‐NKG2A.

We sought to determine if the neutralization of NKG2A could improve the cytotoxic capabilities of such in‐vivo IL‐15 activated NK cells. Neutralization of the NKG2A pathway in mice can be attained in two ways, by blocking NKG2A or blocking its ligand Qa‐1^b^. First, we found that the surface expression of Qa‐1^b^ on the cultured mouse tumor cell lines MC38 and B16.OVA was weak in steady‐state conditions (Appendix Fig S2A and B). However, as expected, the culture of tumor cells in the presence of IFNγ resulted in a marked upregulation of Qa‐1^b^ surface expression (Appendix Fig S2A and B). Indeed, when these cells were co‐cultured with activated NK cells in a 4 h CFSE‐based killing assay, we found that the dual blockade with mAbs directed to NKG2A on the NK side and to Qa‐1^b^ on the tumor side increased the killing of IFNγ‐pretreated tumor cells as compared to isotype‐matched control antibodies. Of note, cytotoxicity was further increased if NKG2A and Qa‐1^b^ were simultaneously neutralized during the assay (Appendix Fig S2C).

**Figure 1:**
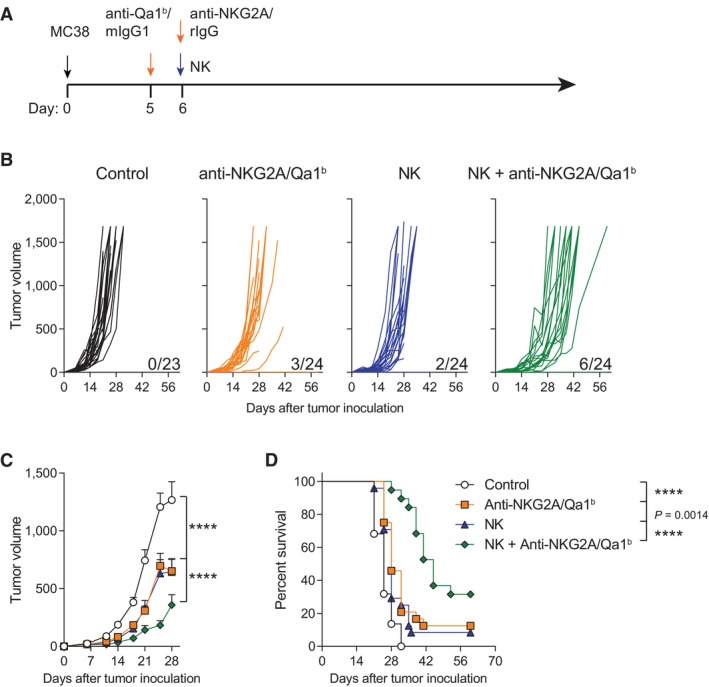
Intratumoral treatment with activated NK cells synergizes with local co‐delivery of anti‐NKG2A and Qa‐1^b^ mAbs A–DC57BL/6 mice bearing MC38 subcutaneous tumors were intratumorally treated with anti‐Qa‐1^b^, anti‐NKG2A or syngeneic donor‐derived activated NK cells at the indicated time points. Control mice and NK cell‐treated mice received mIgG1 and rIgG. (A) Schematic representation of the time course and regimen followed for each treatment. (B) The tumor growth (mm^3^) is shown for each individual tumor. The numbers under each graph represent the fraction of mice that achieved complete tumor regression for each treatment. (C) The average of tumor sizes is shown. (D) The percentage of survival over time is shown. Data information: Data represent four independent experiments with 5–6 mice per group (mean ± SEM). In (C), data were fitted to a third‐order polynomial and compared using extra sum‐of‐squares *F* test. In (D), log‐rank test were used to assess significance. Significant differences are displayed for comparisons of each group with the NK + anti‐NKG2A/Qa‐1^b^ group (****P < 0.0001). Source data are available online for this figure.

**Figure EV1:**
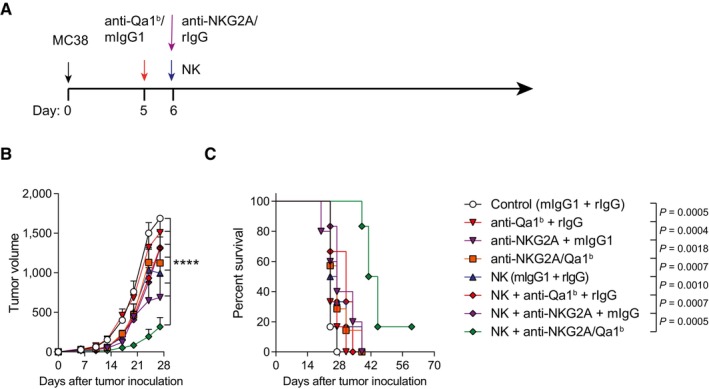
The dual blockade of Qa‐1b and NKG2A within the tumor site better controls tumor growth when combined with activated NK cell intratumoral administration A–C(A) Schematic representation of the regimens followed. mIgG1 and/or rIgG isotype controls were injected when corresponding. (B) The tumor volume (mm^3^) progression is shown over time for each treatment condition (mean ± SEM). (C) The percentage of survival of the indicated groups is shown. Data information: Data are representative of three independent experiments with six mice per group (mean ± SEM). In (B), data were fitted to a third‐order polynomial and compared using extra sum‐of‐squares *F* test. In (D), log‐rank tests were used to assess significance. Significant differences are displayed for comparisons of each group with the NK + anti‐NKG2A/Qa‐1^b^ group (****P < 0.0001).

**Figure EV2:**
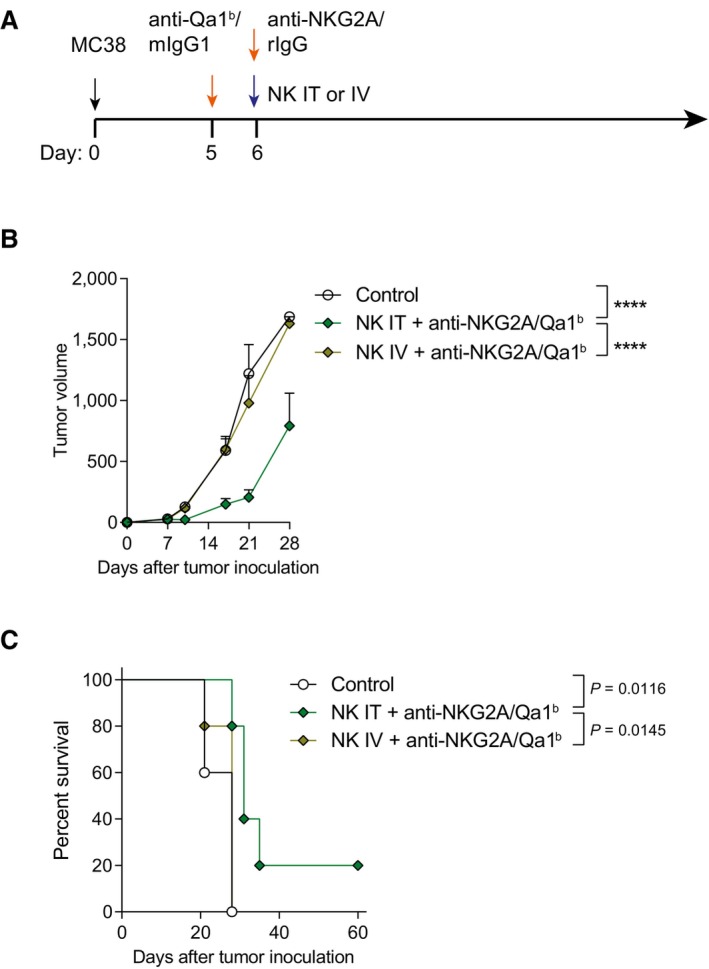
The synergy between anti‐NKG2A/Qa1^b^ and NK cells is only detectable when NK cells are given intratumorally, but not intravenously A–C(A) Schematic representation of the tumor treatment regimen followed in which mice received intratumoral (IT) or intravenous (IV) treatment. Control mice were treated with control mIgG1 and rIgG when indicated. (B) Tumor volume (mm^3^) is shown for each treatment condition. (C) The percentage of survival is shown. Data information: Data represent an experiment with five mice per group (mean ± SEM). In (B), data were fitted to a third‐order polynomial and compared using extra sum‐of‐squares *F* test. In (C), log‐rank tests were used to assess significance (****P < 0.0001).

We next studied if the local dual blockade of NKG2A and Qa‐1^b^ could improve the *in vivo* anti‐tumor efficacy of intratumorally administered NK cells in established mouse tumor models. Anti‐Qa‐1^b^ was injected into the tumor 1 day prior to the intratumoral release of NK cells that were co‐administrated with the anti‐NKG2A mAb (Fig 1A). Each component of the therapy used alone led to a slight delay in tumor growth (Fig 1). In contrast, when the combination of anti‐NKG2A/Qa‐1^b^ and NK cells were intratumorally administered, there was a marked and significant delay of the tumor growth (Fig 1B and C). This delay in tumor growth was also accompanied by an increase in the percentage of tumor‐free surviving mice with a complete rejection of tumors in 25 percent of the treated‐mice (Fig 1D). Of note, optimum control of tumor growth was achieved when NK cells were injected in conjunction with the dual combination of anti‐NKG2A/Qa‐1^b^ mAbs, but not when combined with anti‐Qa‐1^b^ or anti‐NKG2A separately (Fig EV1). Additionally, it is worth mention that the NK cell‐delivery route was important to reach a meaningful control of tumor growth since only when NK cells were given intratumorally, but not intravenously, a therapeutic effect was seen (Fig EV2).

**Figure EV3:**
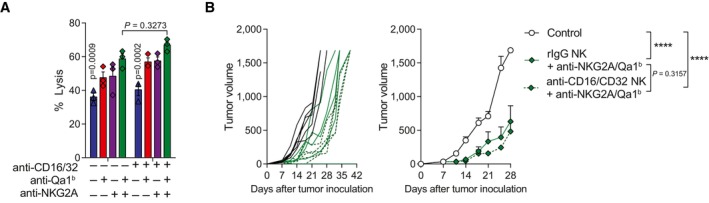
Anti‐Qa‐1^b^‐mediated ADCC does not play a role in the NK and anti‐NKG2A/Qa‐1^b^ immunotherapeutic efficacy A, BActivated NK cells were incubated with anti‐CD16/CD32 or control isotype prior to evaluating their cytotoxicity *in vitro* in a standard 4 h CFSE‐based killing assay. (A) The percentage of tumor lysis is shown for each condition at 5:1 E:T ratio. Isotype control or anti‐CD16/CD32 pretreated NK cells were intratumorally injected following the regimen schedule described in Fig 2A. (B) Tumor growth is shown over time for each individual mice (left panel) or treatment group (right panel). Data information: Data are representative of two independent experiments performed in triplicate (A) or with six mice per group (B) (mean ± SEM). Two‐way ANOVA (A) or extra sum‐of‐squares *F* test of the data fitted to a third‐order polynomial (B) were used to assess significance (****P < 0.0001).

Conceivably, the better anti‐tumor response upon the dual blockade could be due to ADCC in the presence of anti‐Qa1^b^. However, the contribution of ADCC to the overall effect of anti‐NKG2A/Qa1^b^ immunotherapy was considered unlikely since anti‐Qa‐1^b^ mAb is a mouse IgG1, an IgG subclass known to bind to FcγRII and FcγRIII to a lower degree than other IgG subclasses (Stewart *et al*, 2014). To rule out an ADCC effect on the anti‐tumor activity, NK cells were pre‐incubated with anti‐CD16/CD32 mAb to block Fc‐receptor binding prior to the NK‐cell cytotoxicity assays and *in vivo* experiments. As expected, Fc‐blockade with anti‐CD16/CD32 mAb did not alter the performance of NK cells either *in vitro* or *in vivo* (Fig EV3).

**Figure EV4:**
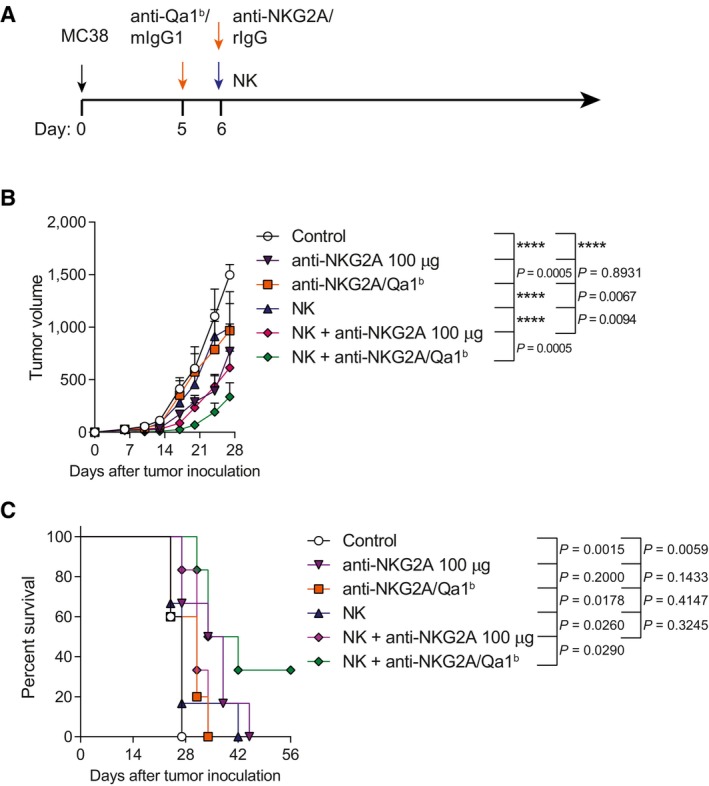
The intratumoral injections of low dose anti‐NKG2A and anti‐Qa1^b^ are more effective than a high dose of anti‐NKG2A ASchematic representation of the dose regimen followed. Control mice and NK cell‐treated mice intratumorally received mIgG1 and rIgG at the same dose as a control.B, CThe average tumor volume (mm^3^) for average tumor progression (B) and the percentage of survival (C) are shown over time. Data information: Data represent an experiment with 5–6 mice per group (mean ± SEM). In (B), data were fitted to a third‐order polynomial and compared using extra sum‐of‐squares *F* test. In (C), log‐rank tests were used to assess significance. Significant differences are displayed for comparisons of each group with the NK + anti‐NKG2A/Qa‐1^b^ group or NK + anti‐NKG2A 100 μg (****P < 0.0001).

To exclude that the greater efficacy of the combination of mAbs was not simply due to a dose effect, we gave anti‐NKG2A mAb at 100 μg, but still the combination of anti‐NKG2A (30 μg) + anti‐Qa1^b^ (30 μg) attained better tumor control than the higher dose of anti‐NKG2A in the same experimental setting (Fig EV4).

### The treatment is equally effective with syngeneic and allogeneic NK cells

**Figure 2:**
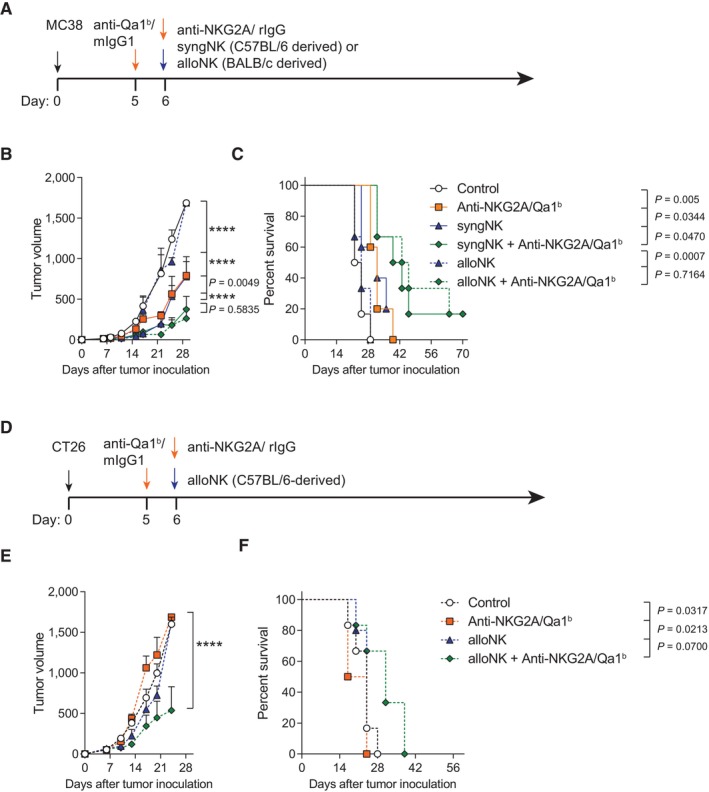
Intratumoral treatment with syngeneic or allogeneic activated NK cells attain comparable anti‐tumor efficacy when the NKG2A pathway is neutralized A–FC57BL/6 mice bearing MC38 subcutaneous tumors were treated with syngeneic C57BL/6‐derived donor NK cells (syngNK) or allogeneic BALB/c‐derived donor NK cells (alloNK). (A) Schematic representation of the regimen followed. Control mice and NK cell‐treated mice received mIgG1 and rIgG. (B) The mean (± SEM) of tumor size volume (mm^3^) for *in vivo* tumor progression is shown for each treated group. (C) The percentage of survival over time is shown. (D) BALB/c mice were inoculated with CT26 subcutaneously and injected with allogeneic C57BL/6‐derived NK cells with or without anti‐NKG2A/Qa‐1^b^ mAbs. (E) The tumor volume (mm^3^) progression is shown over time for each treatment condition (mean ± SEM). (F) The percentage of survival is shown. Data information: Data are representative of two independent experiments with 5–6 mice per group (mean ± SEM). In (B) and (E), data were fitted to a third‐order polynomial and compared using extra sum‐of‐squares *F* test. In (C) and (F), log‐rank tests were used to assess significance. Significant differences are displayed for comparisons of each group with the NK + anti‐NKG2A/Qa‐1^b^ group (****P < 0.0001). Source data are available online for this figure.

In the clinic, there are some scenarios where obtaining patient‐derived NK cells is not possible and the use of donor‐derived NK cells might be a better option. When the efficacy of BALB/c‐derived allogeneic NK cells was evaluated in C57BL/6 mice bearing MC38 tumors, we found that the intratumoral administration of allogeneic NK cells significantly slowed tumor growth when combined with the blockade of NKG2A and Qa‐1^b^. This effect was comparable to that exerted by syngeneic NK cells (Fig 2A and B). The survival benefit was also comparable (Fig 2C). Testing another tumor model, we found that the administration of allogeneic C57BL/6‐derived NK cells together with anti‐NKG2A/Qa‐1^b^ mAbs led to control of tumor progression and enhanced survival of BALB/c mice engrafted with the CT26 colon cancer cells to form established subcutenous tumors (Fig 2D–F). These results indicate that the therapeutic efficacy of combining NK cells and anti‐NKG2A/Qa‐1^b^ is not restricted to a particular mouse strain or to a tumor cell line, and support the feasible use of donor‐derived allogeneic NK cells.

### CD8 T lymphocytes and cDC1 cells underlie the therapeutic efficacy

**Figure 3:**
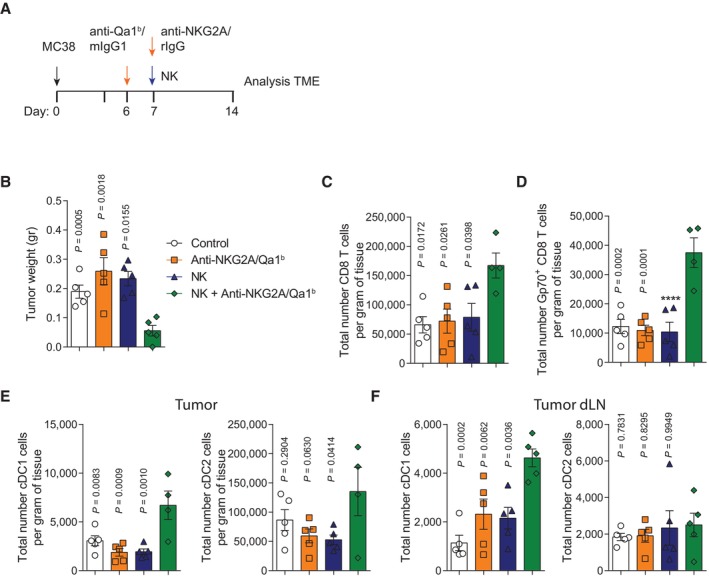
Antigen‐specific CD8 T‐cell and cDC1 numbers are increased in the TME of tumors injected with NK cells and anti‐NKG2A/Qa‐1^b^ mAbs A–FC57BL/6 mice were subcutaneously injected with MC38 tumors and treated according to the regimen described in Fig 1. Organs were collected at day 14 post‐tumor inoculation and the immune cell compartment was evaluated by multiparametric flow cytometry. (A) Schematic representation of the time course and the treatment regimens followed. Control mice and NK cell‐treated mice intratumorally received mIgG1 and rIgG at the same dose as a control. (B) Tumor weight (gr.) is shown for each treatment at day +14. (C) The total number of CD8 T cells (CD45.2^+^CD19^−^TCRβ^+^NK1.1^−^CD4^−^CD8^+^) per gram of tissue is shown. (D) The total number of Gp70‐reactive CD8 T cells detected by MHC pentamer staining is shown. (E, F) The total number of cDC1 (CD45.2^+^CD19^−^F4/80^−^TCRβ^−^NK1.1^−^MHCII^+^CD11c^+^CD11b^−^) and cDC2 (CD45.2^+^CD19^−^F4/80^−^TCRβ^−^NK1.1^−^MHCII^+^CD11c^+^CD11b^+^) cells is shown for treated tumors (E) and tumor dLNs (F). Data information: Data are representative of two independent experiments with five or six mice per group (mean ± SEM). One‐way ANOVA was used to determine statistical significance. Significant differences are displayed for comparisons of each group with the NK + anti‐NKG2A/Qa‐1^b^ group (****P < 0.0001). Source data are available online for this figure.

To study the effects of treatment in the tumor tissue microenvironment and in dLNs, cell suspensions of these tissues were analyzed by flow cytometry 8 days post‐treatment (Fig 3A). A marked reduction in tumor weight was associated with NK and anti‐NKG2A/Qa‐1^b^ treatment when compared to treatments of control isotype, or single anti‐NKG2A/Qa‐1b mAbs or NK cells (Fig 3B). The analysis of the tumors showed a significant increase in total CD8 T cells and tumor antigen‐specific Gp‐70‐pentamer^+^ CD8 T cells in the combination treatment group as compared to controls treated with isotype‐matched irrelevant mAbs and mice receiving a single treatment with anti‐NKG2A/Qa‐1^b^ or with NK cells (Fig 3C and D). We also observed that the degranulation capacity of tumor‐infiltrating CD8 T cells was increased on mice intratumorally treated with anti‐NKG2A/Qa1^b^ and/or NK cells when compared to isotype control‐treated mice (Appendix Fig S3A–C). However, no significant functional differences were observed among tumor‐infiltrating CD8 T cells among treatment groups. In addition, the percentage of CD8 T cells producing IFNγ was not increased in any condition on per‐cell basis (Appendix Fig S3D and E). These results indicate that the anti‐tumoral efficacy of the NK + anti‐NKG2A/Qa1^b^ treatment is likely due to an increase in the absolute number of antigen‐specific CD8 T cells, rather than to an increase of CD8 T‐cell functional capacities on per‐cell basis.

Interestingly, an increase in cDC1 cells was also observed in both tumor nodules and tumor dLNs (Fig 3E and F). In contrast, cDC2 cell numbers were only slightly elevated in the tumors upon treatment, but not in the tumor dLNs (Fig 3E and F).

The NK‐cell compartment was also slightly, although not significantly, increased in the TME of the NK and anti‐NKG2A/Qa‐1^b^‐treated groups (Appendix Fig S4A), even if such NK cells were proliferating more avidly according to Ki67 expression (Appendix Fig S4B). This increase in NK cells could be the result of the exogenous administration of syngeneic NK cells. However, it is well‐known that activated NK cells undergo apoptosis shortly after injections if no exogenous cytokines such as IL‐2, IL‐7 or IL‐15 are given (Ochoa *et al*, 2013; Alvarez *et al*, 2019). The use of congenic CD45.1 NK cells as a source for intratumoral NK cell therapy allowed us to investigate the presence of endogenous (CD45.2^+^) and exogenous (CD45.2^−^) NK cells in the TME 4 days after NK‐cell intratumoral delivery (Appendix Fig S4C). As expected, at this time point, < 5% of the total NK cells found in the TME were of donor‐origin (Appendix Fig S4D). These data reinforce the short time‐span of exogenously injected NK cells and, importantly, that endogenous NK cells are recruited to the tumor microenvironment.

**Figure 4:**
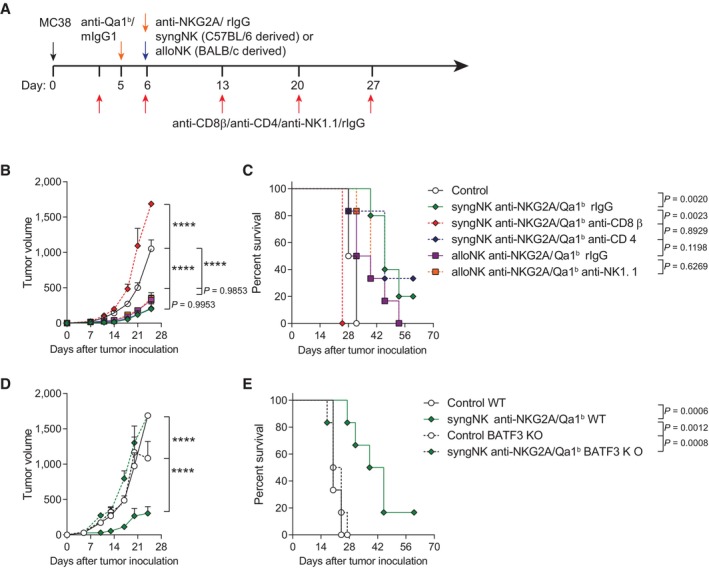
CD8^+^ T cells and cDC1 cells play a critical role in the efficacy of the combined intratumoral treatment A–EWT or BAFT3‐deficient C57BL/6 tumor‐bearing mice were treated with anti‐Qa‐1^b^, anti‐NKG2A and/or syngeneic (C57BL/6‐derived) and allogeneic (BALB/c‐derived) NK cells as in Fig 2. When indicated, mice received intraperitoneal injections of anti‐CD8β, anti‐CD4 or anti‐NK1.1 to deplete endogenous CD8 T, CD4 T or NK cells. (A) Schematic representation of the time course and treatment regimens followed. Control mice and NK cell‐treated mice intratumorally received mIgG1 and rIgG at the same dose as a control. (B, C) The average tumor volume (B) for *in vivo* tumor progression and the percentage of survival (C) are shown over time. (D, E) The average tumor growth (mm^3^) and the percentage of survival are shown for BATF 3 KO mice and their WT counterparts after NK and anti‐NKG2A/Qa‐1^b^ intratumoral treatment. Data information: Data are representative of two independent experiments with five or six mice per group (mean ± SEM). One‐Way ANOVA was used to determine statistical significance. In (B) and (D), data were fitted to a third‐order polynomial and compared using extra sum‐of‐squares *F* test. In (C) and (E), log‐rank test was used to assess significance. Significant differences are displayed for comparisons of each group with the NK (syngeneic or allogeneic) + anti‐NKG2A/Qa‐1^b^ group (****P < 0.0001). Source data are available online for this figure.

We next performed depletion experiments to selectively eliminate CD8 T (anti‐CD8β), CD4 T (anti‐CD4) and NK cells (anti‐NK1.1; Fig 4A). To only eliminate endogenous NK cells, we used allogenic BALB/c‐derived NK cells for the NK cell‐depletion experiments, for the reason that BALB/c NK cells do not express the NK1.1 allele. Depletion studies demonstrated that only when CD8 T cells were eliminated, efficacy was lost (Fig 4B and C). Hence, a necessary role of CD4 T cells and endogenous NK cells can be excluded (Fig 4B and C).

To determine if the binding of free anti‐NKG2A on tumor infiltrating CD8 T cells could account for the anti‐tumor response mediated by NK + anti‐NKG2A/Qa1^b^, we performed an experiment where NK cells were pre‐incubated with anti‐NKG2A or isotype control, and the free mAb was washed off prior to the intratumoral injection of NK cells (Appendix Fig S5A). In this scenario, the anti‐tumor response mediated by anti‐NKG2A pre‐coated NK cells + anti‐Qa1^b^ was comparable to the efficacy results achieved by NK cells + anti‐NKG2A/Qa1b (Appendix Fig S5B and C); therefore suggesting the effect of anti‐NKG2A mAb is primarily exerted on the injected NK cells. In recent work done by our group (Minute *et al*, 2020), we revealed an increase on the antigen uptake capabilities by cDC1 cells exposed to tumor cell debris from NK cell‐killed tumor cells (Minute *et al*, 2020). Importantly, upon injection of tumor cells that had been killed in culture by NK cells, there was an induction of specific CD8 T‐cell responses in the mice. Such an effect required BATF3‐dependent cDC1 dendritic cells that cross‐present tumor antigens. Consequently, as a logical next step, the anti‐tumor efficacy of intratumoral NK and anti‐NKG2A/Qa‐1^b^ therapy was evaluated in BATF3^−/−^ mice which are devoid of cDC1 cells (Hildner *et al*, 2008). As expected, treatment showed no signs of efficacy in cDC1‐deficient mice (BATF3 KO) as compared to wild‐type mice (Fig 4D and E). This is explained by the key role of cDC1 cells orchestrating CD8 T‐cell responses including their necessary role for tumor antigen cross‐priming (Theisen *et al*, 2018). Minute L. and collaborators proposed that the cytotoxic immune response obtained by NK cell‐mediated tumor lysis could unleash immunogenic cell death (ICD) and orchestrate the antigen‐specific CD8 T‐cell response required for tumor control (Galluzzi *et al*, 2020; Minute *et al*, 2020). In line with this study, we found that cell suspensions from excised MC38 malignant cells from intratumorally treated mice (Appendix Fig S6A) displayed on the plasma membrane higher levels of the pro‐phagocytic molecule calreticulin, whose expression was significantly brighter in the tumors that were treated with NK + anti‐NKG2A/Qa1^b^ (Appendix Fig S6B and C). Interestingly, activated caspase‐3 was also significantly enhanced in tumor cells from intratumorally NK + anti‐NKG2A/Qa1^b^‐treated mice (Appendix Fig S6D and E). These data indicate the occurrence of ICD following intratumoral NK +/− anti‐NKG2A/Qa1^b^ therapy.

### Systemic anti‐PD‐1 therapy synergizes with local delivery of NK cells plus anti‐NKG2A/Qa‐1^b^ mAb therapy

**Figure 5:**
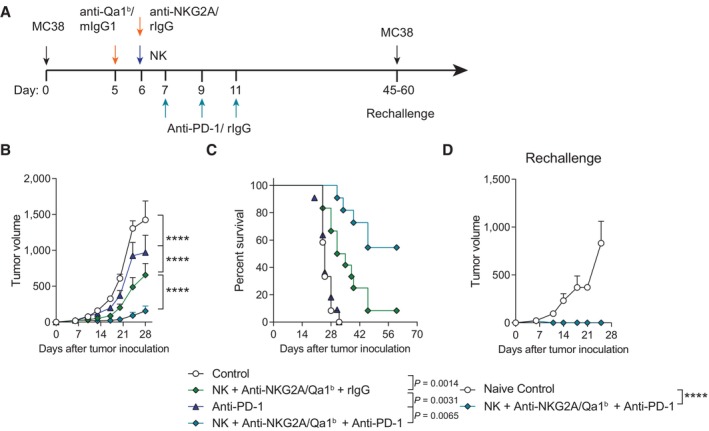
Concomitant systemic blockade of PD‐1 in a PD‐1‐resistant model improves the anti‐tumor effects attained by the intratumoral co‐injections of activated NK cells and anti‐NKG2A/Qa‐1^b^ mAbs A–D(A) Schematic representation of the time course and treatments regimens used. Control mice and NK cell‐treated mice intratumorally received mIgG1 and rIgG at the same dose as a control. (B) The mean (± SEM) of tumor size volume (mm^3^) for *in vivo* tumor progression is shown for each treatment group. (C) The percentage of survival is shown over time. (D) Surviving mice from two independent experiments (seven mice in total) from C were rechallenged on days 45–60 with MC38 subcutaneously. Tumor growth (mm^3^) progression of MC38 is shown for naïve resting control mice and the surviving mice from experiments of C. Data information: Data represents two independent experiments with five or six mice per group (mean ± SEM). In (B) and (D), data were fitted to a third‐order polynomial and compared using extra sum‐of‐squares *F* test. In (C), log‐rank tests were used to assess significance. Significant differences are displayed for comparisons of each group with the NK + anti‐NKG2A/Qa‐1^b^ group (B, C) or NK + anti‐NKG2A/Qa‐1^b^ + anti‐PD‐1 group (D) (****P < 0.0001). Source data are available online for this figure.

**Figure EV5:**
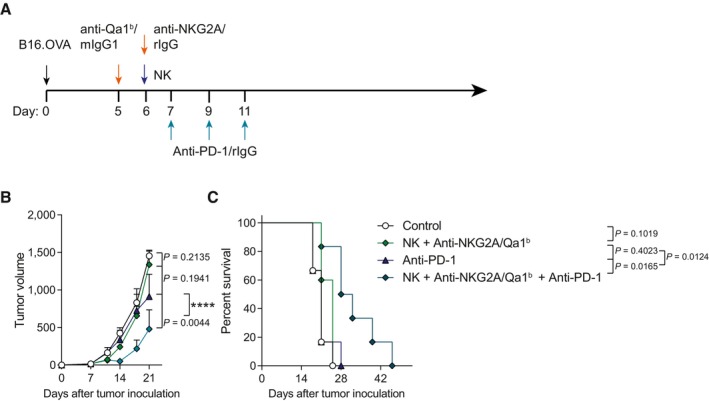
Anti‐PD‐1 therapy synergizes with intratumoral NK and anti‐NKG2A/Qa‐1^b^ therapy to better control the growth of B16.OVA melanoma A–C(A) Schematic representation of the regimes followed. Control mice and NK cell‐treated mice received mIgG1 and rIgG. (B) The means of tumor size (mm^3^) is shown over time for each treatment condition. (C) The percentage of survival as shown in a Kaplan–Meier graph. Data information: Data are representative of two independent experiments with six mice per group. Extra sum‐of‐squares *F* test of the data fitted to a third‐order polynomial (B) or log‐rank tests were used to assess significance. Significant differences are displayed for comparisons of each group with the NK + anti‐NKG2A/Qa‐1^b^ group or the NK + anti‐NKG2A/Qa‐1^b^ + anti‐PD‐1 group (****P < 0.0001).

We next studied if the intratumoral NK cells plus an anti‐NKG2A/Qa‐1^b^ combination with immune checkpoint blockade (ICB) therapy could further increase therapeutic efficacy. Among its mechanisms of action, ICB has proven to revert CD8 T‐cell exhaustion (Siddiqui *et al*, 2019). The role of CD8 T‐cell efficacy advocated for combinations with ICB. Supporting this notion, we found that the anti‐tumor response mediated by intratumoral injections of anti‐NKG2A/Qa‐1^b^ plus activated NK cells was greatly improved by the administration of intraperitoneal anti‐PD‐1 mAb (Fig 5A–C). This triple combination not only delayed the progression of tumor growth (Fig 5B) but also markedly increased the percentage of mice that completely rejected their tumors due to treatment, as compared with mice receiving intratumoral treatment and an irrelevant intraperitoneal control mAb (Fig 5C). Similar results were obtained when the combined therapy was administered into B16.OVA‐derived tumors, which are more resistant to NK cell‐killing given the lack of expression of NKG2D ligands (Fig EV5; Alvarez *et al*, 2019). The mice that underwent complete tumor regression in Fig 5C were then rechallenged with the same tumor (MC38) in the right flank 45–60 days after primary tumor inoculation (Fig 5A). Importantly, all the mice that had rejected MC38 tumors as a result of the intratumoral combined therapy were resistant to MC38 rechallenge in contrast to naïve control mice (Fig 5D).

**Figure 6:**
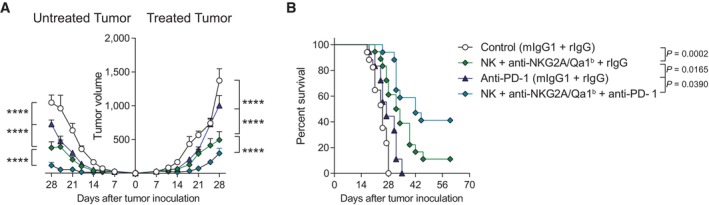
Local and abscopal anti‐tumor effects elicited by intratumoral NK cells and anti‐NKG2A/Qa‐1^b^ in combination with systemic PD‐1 blockade A, BC57BL/6 mice bearing two MC38 tumors subcutaneously engrafted in opposite flanks received treatment in one tumor following the schedule regimen described in Fig 5A, while the distant tumor was left untreated. (A) The progression for the tumor volume (mm^3^) is shown over time for treated and untreated tumors. (B) The percentage of mouse survival is shown. Data information: Data represent three independent experiments with five or six mice per group (mean ± SEM). Extra sum‐of‐squares *F* test of the data fitted to a third‐order polynomial (A) or log‐rank tests (B) were used to determine statistical significance. Significant differences are displayed for comparisons of each group with the NK + anti‐NKG2A/Qa‐1^b^ group (****P < 0.0001). Source data are available online for this figure.

Next, we investigated the ability of the intratumoral NK and anti‐NKG2A/Qa‐1^b^ mAb therapy to exert systemic responses against concomitant tumors not receiving direct intratumoral treatment. In a bilateral tumor mouse model in which only one of the tumors received treatment, the NK + anti‐NKG2A/Qa‐1^b^ injections led to a significant delay in tumor growth in both treated and untreated tumors (Fig 6A) with an increase in the survival levels when compared to control mice (Fig 6B). Importantly, in these settings, the systemic administration of anti‐PD‐1 mAb along with intratumoral NK + anti‐NKG2A/Qa‐1^b^ mAb therapy to only one of the tumors was capable of better controlling tumor growth in both the treated and the distant tumors (Fig 6A), significantly improving survival (Fig 6B). These results demonstrate the existence of a synergistic effect when systemic anti anti‐PD‐1 mAb therapy is combined with the local intervention.

### Combined intratumoral injections of monalizumab and human NK cells against xenografted human tumors in immunodeficient mice

**Figure 7:**
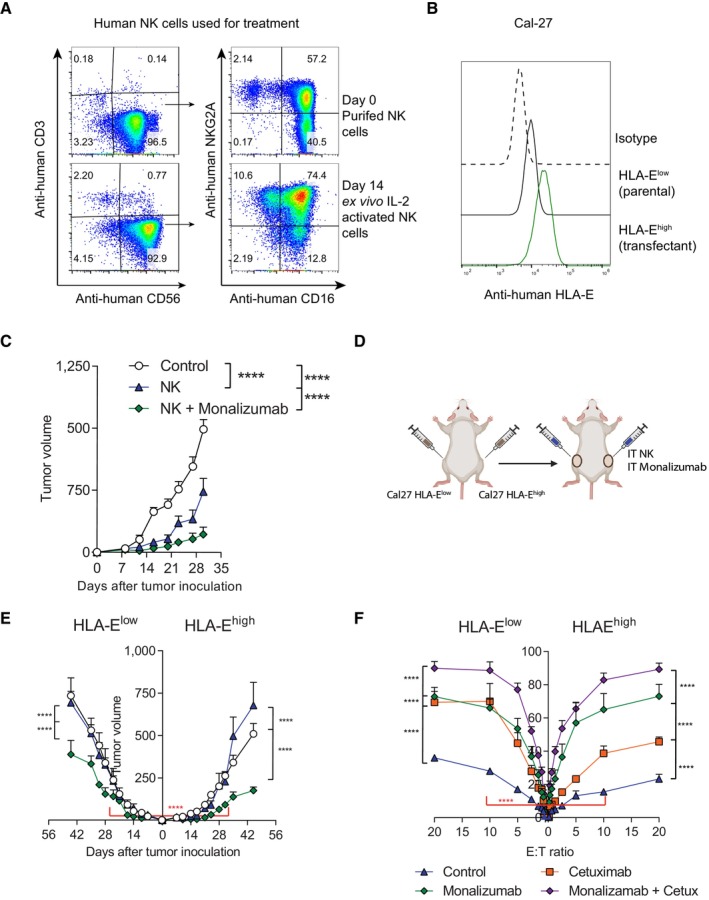
Intratumoral adoptive transfer of *ex vivo* expanded human NK cells co‐injected with monalizumab synergize to control growth of xenografted HLA‐E^+^ tumors in immunodeficient mice A, B(A) Representative dot‐plots of purified human NK cells (CD3^−^CD56^+^) and their corresponding surface expression of CD16 and NKG2A prior (day 0) and after *ex vivo* IL‐2‐driven NK cell expansion (day 14). (B) Representative histogram of HLA‐E expression for parental (HLA‐E^low^) and HLA‐E‐transfected (HLA‐E^high^) Cal‐27 tumor cell line.CCal‐27 tumor‐bearing Rag2^−/‐^IL2Rγ^−/−^ immunodeficiency mice were intratumorally treated with activated NK cells and monalizumab. The mean (± SEM) of tumor size volume (mm^3^) is shown over time for each treated group.D, E(D) As indicated in the scheme, HLA‐E^low^ and HLA‐E^high^ Cal‐27 tumor cells were implanted on opposite flanks of Rag2^−/‐^IL2Rγ^−/−^ mice and both tumors were intratumorally treated with NK cells and monalizumab. (E) The mean (mm^3^) of tumor volume progression is shown over time for each treatment condition for Cal‐27 HLA‐E^low^ and HLA‐^high^ Cal‐27 tumor‐bearing mice.FThe indicated tumor cells were co‐cultured *in vitro* with NK cells and the tumor lyses was assessed in standard 4 h Cr^51^ release assays. The percentage of lyses of HLA‐E^low^ Cal‐27 (left) and HLA‐E^high^ Cal‐27 (right) tumor cells are shown for each treatment condition at different effector‐target (E:T) ratios. Data information: Data show on (C) and (E) represents two independent experiments with six mice per group (mean ± SEM) with NK cells obtained from one healthy donor for each experiment. Data shown on F represent the reproductive results of one out of two independent experiments done in triplicate with NK cells obtained from two healthy donors for each experiment. Extra sum‐of‐squares *F* test of the data fitted to a third‐order polynomial were used to determine statistical significance for (C), (E) and (F). Significant differences are displayed for comparisons of each group with the NK + monalizumab group (****P < 0.0001). Red color indicates the significant differences for NK + monalizumab or NK cell groups between HLA‐E^low^ and HLA‐E^high^ CAL‐27 tumors for (E) and (F), respectively. Source data are available online for this figure.

To address if the effects observed in mice could be extended to humans, we evaluated the therapeutic efficacy of intratumoral injections of activated NK cells combined with the anti‐NKG2A mAb monalizumab into xenografted human tumors. This model allowed to study if the direct NK‐cell lysis of tumor cells can be enhanced by monalizumab co‐treatment in an *in vivo* setting when these agents are given locally into the tumors. Purified NK cells from PBMCs were *ex vivo* expanded for 14 days with rhIL‐2 (Fig 7A). As in the case of mouse NK cells, human NK cells also upregulated NKG2A expression upon activation (Fig 7A). Because the human tumor cell lines that we analyzed weakly expressed surface HLA‐E, we transfected the squamous cell carcinoma CAL‐27 cell line, whose expression of HLA‐E was at baseline around 20%, with a plasmid encoding the HLA‐E plus the HLA‐G signal peptide to achieve HLA‐E surface expression (Fig 7B).

To demonstrate the *in vivo* efficacy of monalizumab in conjunction with intratumoral delivery of human NK cells, transfected HLA‐E^high^ tumor cells were subcutaneously xenografted in Rag2^−/‐^IL2Rγ^−/−^ immunodeficient mice and 7 days later when tumors reached approximately 40 mm^3^, mice were intratumorally co‐injected with activated human NK cells with or without monalizumab or control antibody. The intratumoral co‐injection of monalizumab markedly reduced tumor growth in the NK cell‐treated mice (Fig 7C).

To determine the influence of the level of HLA‐E surface expression on the malignant cells, we inoculated HLA‐E^low^ (non‐transfected) and HLA‐E^high^ (transfected) Cal‐27 tumor‐cell variants on opposite flanks of Rag2^−/‐^IL2Rγ^−/−^ mice (Fig 7D). As expected, monalizumab significantly improved the anti‐tumor effect elicited by the intratumoral release of NK cells, more evidently in the HLA‐E^high^ tumors (Fig 7E). A previous study had shown that NK cell‐mediated ADCC induced by cetuximab was enhanced by monalizumab (Andre *et al*, 2018). Thus, a standard Cr^51^‐release killing assay on parental (HLA‐E^low^) and transfected (HLA‐E^high^) CAL‐27 tumor cells confirmed that monalizumab enhanced NK‐cell killing, to a larger extent in the case of HLA‐E‐expressing tumors (Fig 7F). Notably, the blockade of NKG2A on NK cells with monalizumab significantly increased the lysis percentage of Cal‐27 tumors co‐cultured with cetuximab to elicit ADCC (Fig 7F). The influence of HLA‐E tumor expression levels in susceptibility to treatment may suggest its value as a biomarker.

## Discussion

The ability to mediate cytotoxicity is highly restricted to activated T and NK lymphocytes. Such a function, that is necessary for antiviral defense and anti‐tumor immunosurveillance, is dangerous and needs to be under tight control. When considering adoptive transfer of cytotoxic lymphocytes, tampering with their regulatory mechanisms is to be considered either systemically or locally (Muntasell *et al*, 2017; Cherkassky *et al*, 2022).

In this study, we report on an immunotherapy strategy based on intratumoral delivery of activated NK cells. Such an approach has been tested previously and only modest effects were attained in tumor‐bearing mice (Ishikawa *et al*, 2004; Capobianco *et al*, 2008), as we have confirmed in our experiments when using either autologous or allogeneic mouse NK cells. This is not surprising since even if effector NK cells would efficiently kill tumor cells, the overwhelming number of malignant cells in a tumor lesion surpasses the ability of NK lymphocytes to kill such large number of targets. This is in spite of the fact that properly performed local delivery bypasses the need for trafficking into the tumor tissue, which is reportedly an obstacle for NK‐based immunotherapies (Melero *et al*, 2014).

In accordance with the missing‐self hypothesis (Kärre, 2002), MHC‐I‐negative tumor cells are more suitable for NK cytotoxicity, either spontaneous or ADCC. This is due to the fact that human and mouse NK cells express inhibitory receptors on their surface that recognize MHC‐I molecules. KIRs in humans and Ly49 molecules in mice mediate such a function in a MHC‐I allele‐specific manner (Borrego, 2006). An alternative mechanism is mediated by NKG2A/CD94 recognition of the non‐classical MHC‐I proteins HLA‐E in humans and Qa‐1^b^ in mice, whose presence on the membrane is contingent on the concomitant expression of the leader sequence of classical MHC‐I molecules that provide peptides to stabilize HLA‐E and Qa‐1^b^ in humans and mice, respectively (Lee *et al*, 1998a). The inhibitory function of NKG2A is conserved in rodents and primates and amenable to being blocked by mAbs. Of note, NKG2A is also expressed and functional in a fraction of αβ and γδ T cells where it also controls cytotoxicity (van Hall *et al*, 2019; Borst *et al*, 2020). The importance of this inhibitory pathway is reflected by the established association of HLA‐E expression with poor prognosis in series of solid and hematological cancers (van Montfoort *et al*, 2018; Hiraoka *et al*, 2020; Wu *et al*, 2020). Conceivably, the NKG2A pathway represents a promising target to enhance NK and/or T cell function in the TME (Andre *et al*, 2018; van Montfoort *et al*, 2018).

Hence, we sought to co‐inject blockers of the NKG2A inhibitory pathway together with autologous or allogeneic NK cells in mice. As a result of the combination, more prominent anti‐tumor activity was observed. In our study, the intratumoral combination of anti‐NKG2A plus anti‐Qa1^b^ mAbs empirically performed better than single mAbs for unknown reasons that do not simply involved intratumoral concentrations or ADCC. In these treatment conditions, intratumorally delivered NK cells may directly curtail tumor progression as a direct result of more efficient killing. However, the intratumoral treatment may also act as a starting mechanism for CD8 T‐cell responses (Melero *et al*, 2021). In this regard, it is known that the IFNγ produced by NK cells induces chemokines that attract CD8 T cells, such as CXCL9 and CXCL10. Intratumoral NK cells also favor the activity and recruitment of cDC1 cells, specialized in antigen cross‐priming to CD8 T lymphocytes (Barry *et al*, 2018; Böttcher *et al*, 2018). Our experiments clearly showed that both tumor‐specific CD8^+^ T cells, cDC1 and endogenous NK cells are driven into the tumors as a result of treatment. More importantly, CD8 T‐cell depleted and cDC1‐deficient BATF3^−/−^ mice do not benefit at all from the combined intratumoral treatment. These findings are consistent with observations on NK‐cell cytotoxicity being a form of immunogenic cell death that provides antigenic material for T cell‐cross‐priming and results in anti‐tumor CD8 T‐cell responses (Mocikat *et al*, 2003; Krebs *et al*, 2009; Pitt *et al*, 2017; Galluzzi *et al*, 2020, 2023; Minute *et al*, 2020). ICD and cytotoxicity mediated by NK and T cells is an important element intended by our treatment strategy. Surface exposure of calreticulin by tumor cells in treated tumors speaks in favor of such phenomena (Pitt *et al*, 2017; Galluzzi *et al*, 2020, 2023). Of note, in a similar fashion, local release of anti‐tumor cytotoxic T cells facilitates the cross‐priming mediated by cDC1 cells (Etxeberria *et al*, 2019). However, the interplay of NK cells and cDC1 has been repeatedly reported in tumors and consider of much importance to regulate CTL cross‐priming (Mocikat *et al*, 2003; Krebs *et al*, 2009; Barry *et al*, 2018; Böttcher *et al*, 2018).

A negative consequence of local IFNγ production by the injected NK cells is PD‐(L)1 upregulation on tumor cells (Dong *et al*, 2002). Cognizant of this factor, we tested the combination of local NK delivery and NKG2A suppression with PD‐1 blockade. Synergistic effects were achieved that were able to control or reject distant untreated tumors in the MC38 model that is completely resistant to PD‐1 blockade. IFNγ also results in enhanced MHC‐I expression and consequently of Qa‐1^b^ and HLA‐E, establishing a rationale for the combination regimen. Recent work has provided evidence for the role of the NKG2A inhibitory pathway in the resistance to anti‐PD‐1 therapy in mice (Zhang *et al*, 2021; Battaglia *et al*, 2022). In line with this, other authors have found that only when Qa‐1^b^ was eliminated from anti‐PD‐1 resistant TAP^−/−^ B16F10 tumors, was the efficacy of anti‐PD‐1 restored (Zhang *et al*, 2021). Further pieces of evidence came from the NKG2A neutralization in tumor models exposed to radiotherapy, which upregulates Qa‐1^b^ expression and compromises the anti‐tumor efficacy mediated by anti‐PD‐1 mAbs (Battaglia *et al*, 2022). It is tempting to speculate as a general principle that adoptive cell therapy with T or NK lymphocytes will benefit from dual concurrent PD‐1 and NKG2A blockade.

Our experiments provide evidence for a strategy of treatment in three experimental mouse models. The question was whether such an approach could be suitable for clinical translation. We believe that this is the case since: (i) NK cells of autologous or allogeneic origin can be isolated and grown under GMP conditions (Lamers‐Kok *et al*, 2022); (ii) local and intracavitary delivery of CAR‐T cells is feasible and is being performed in clinical trials (Cherkassky *et al*, 2022), a concept that could be easily applied to GMP‐cultured NK cells as well; (iii) there are safe clinical grade NKG2A mAbs in clinical trials including monalizumab (van Hall *et al*, 2019; Borst *et al*, 2020); and (iv) the elements and mechanisms observed are conserved between rodents and humans. In our strategy, local delivery of NK cells seems to be crucial for efficacy. In our view, this approach maximizes NK‐cell bioavailability to mediate cytotoxicity as a form of immunogenic cell death. For local image‐guided intratumoral injection in humans, reliable procedures and methods are to be optimized. Regarding co‐administration of the NKG2A neutralizing mAbs, systemic administration is probably preferred, perhaps in combination with PD‐(L)1‐neutralazing agents.

To establish the proof‐of‐concept that this strategy may work in cancer patients, we performed experiments on xenografted human HLA‐E^+^ tumors implanted in immunodeficient mice. Such experiments showed evidence for a greater effect of the combination of local monalizumab when co‐injected with allogeneic NK cells. This is in spite of the fact that in this case no ensuing T‐cell responses could occur in the xenoengrafted immunodeficient mice.

All in all, local delivery of “off‐the‐shelf” NK cells could be an attractive option to control tumor lesions in combination with NKG2A inhibitors that might also be given systemically. This maneuver can result in efficient local priming of tumor‐specific CD8^+^ T cells that are to be de‐repressed by checkpoint inhibitors in triple regimens for cancer immunotherapy, in an attempt to attain synergistic effects in the clinic.

## Material and Methods

### Mice

Female and male 8–12 weeks C57BL/6 or BALB/c mice were purchased from Harlan Laboratories (Barcelona, Spain). Female C57BL/6 Batf3^tm1Kmm^/J (BATF3 KO) (Hildner *et al*, 2008) or wild type counterparts were kindly provided by Dr. Kenneth M. Murphy (Washington University, St. Louis, MO) and bred at the CIMA/University of Navarra animal facility. B6.129S7‐Rag1^tm1Mom^ (B6 Rag1), C.129S7(B6)‐Rag1^tm1Mom^ (BALB/c Rag1), BALB/c‐Rag2^tmlFwa^IL2rg^tmlWjl^ (Rag2^−/−^IL2Rγ^−/−^) deficient mice and B6.SJL‐Ptprc^a^ Pepc^b^/BoyJ (CD45.1 C57BL/6) mice were originally purchased from the Jackson Laboratories (Bar Harbor, ME) and bred at the CIMA animal facility. All animals were group‐housed under specific pathogen‐free conditions and maintained on a 12‐h light–dark cycle. Water and standard laboratory rodent chow diet were available *ad libitum*. The Ethics Committee of Animal Experimentation (ECAE) at the University of Navarra approved the animal protocol (#060‐21).

### Cell lines

C57BL/6‐derived MC38, BALB/c‐derived CT26 mouse colon carcinoma and C57BL/6‐derived B16‐OVA mouse melanoma cell lines were kindly gifted by Dr. Karl E. Hellström (University of Washington, Seattle, WA), Mario Colombo (IRCCS Instituto Nazionale dei Tumori. Milano, Lombardia, Italia) and Dr. Lieping Chen (Yale University, New Heaven, CT), respectively. These cell lines were authenticated by Idexx Radil (Case 6592‐2012). MC38 and CT26 cells were grown in RPMI 1640 media supplemented with GlutaMAX™ (Gibco™), 10% heat‐inactivated FBS, 50 μM 2‐mercaptoethanol, 100 U/ml penicillin, and 100 μg/ml streptomycin at 37°C with 5% CO_2_ (complete media). B16.OVA tumor cells were grown in complete media supplemented with 400 μg/ml Geneticin (Gibco™). Cells were collected for tumor studies when they reach exponential growth during that week of culture. The human Cal‐27 squamous cell carcinoma cell line was purchased from ATCC in 2011 and retrieved from a verified master cell bank. These cells were culture with RPMI 1640 media supplemented with GlutaMAX™ (Gibco™), 10% heat‐inactivated FBS, 100 U/ml penicillin, and 100 μg/ml streptomycin at 37°C with 5% CO_2_. This cell line was not authenticated for this project. All cell lines were tested monthly for mycoplasma contamination (MycoAlert Mycoplasma Detection Kit, Lonza).

### Isolation and *in vivo* activation and expansion of NK cells

NK cells were obtained from the spleens of B6 Rag1 KO mice that 3 days previously received hydrodynamic injections of 10 μg of a plasmid containing a codon‐optimized cDNA that synthesize a fusion recombinant chimeric protein encompassing the sushi domain of the IL15Rα, IL‐15, and apolipoprotein A‐I (Sushi‐IL15‐Apo) as previously described (Ochoa *et al*, 2013). For NK cell isolation, the EasySep™ Mouse NK cell isolation kit was used according to manufacturer's instructions (StemCell Technology, Vancouver, Canada). NK cell purity measured by CD3^−^NK1.1^+^ or CD3^−^CD49b^+^ was determined by flow cytometry as previously described (Alvarez *et al*, 2020a). Briefly, cells were incubated with Fc‐block (anti‐CD16/32) at 4°C prior to incubation with surface mAbs to prevent unspecific binding.

### Flow cytometry analysis

*Analysis of Qa‐1* ^*b*^ *‐expression*: for *in vitro* experiments, MC38 or B16 tumor cells were treated with 600 IU/ml of recombinant mouse Interferon gamma (IFNγ Peprotech, Cranbury, NJ) or corresponding controls. Tumor cells were collected after 48 h of culture, and single‐cell suspensions were stained with biotinylated anti‐mouse Qa‐1^b^ (BD Bioscience, Franklin Lakes, NJ) or mIgG1 isotype control for 20 min at 4°C, followed by staining with streptavidin‐APC for 10 min at 4°C.

*Analysis of NKG2A expression*: purified naïve and IL‐15 activated NK cells were stained with anti‐mouse NKG2A (clone 16A11), anti‐mouse NKG2C (clone 2098A) or the corresponding isotype control. Anti‐mouse CD90.2 (clone 30‐H12) was also included to verify NK cell activation.

*Analysis of the immune cell compartment in the TME and tumor dLNs*: 6 × 10^5^ MC38 cells were inoculated into C57BL/6 mice and the tumors were treated with anti‐Qa‐1^b^ and/or NK cells plus anti‐NKG2A on days six and seven, respectively. Tumors, tumor dLNs and spleens were collected 14 days post‐tumor inoculation, and single cell suspensions were stained as described in the previous sections. The processing of tumors involved the digestion of excised and minced tumors with 400 U/ml of collagenase D and 50 μg/ml of DNase‐I (Roche Basel, Switzerland) for 20 min at 37°C. Then, tumors were mechanically disaggregated and filtered through a 70 μm cell strainer (Thermo Fisher Scientific). Tumor infiltrating lymphocytes (bottom) and tumor cells (top) were then isolated after Percoll 40% (Merck, Darmstadt, Germany) centrifugation. Additionally, the CD8 T‐cell compartment (CD45^+^CD19^−^TCRβ^+^NK1.1^−^CD8^+^CD4^−^) was analyzed for antigen specificity with the gp70 pentamer (ProImmune) following manufacture's instructions. In some experiments, tumor infiltrated lymphocytes were stimulated with complete media supplemented with 160 ηg/ml of phorbol 12‐myristate 13‐acetate (PMA, SIGMA) and 1.6 μg/ml of Ionomycin (SIGMA). The degranulation marker anti‐CD107a (BioLegend) was added at 1:200 dilution at the beginning of the 4 h stimulation. 1 μg/ml monensin (BD, Pharmigen) was added to the media 1 h after adding the stimulation medium. 4 h later, cells were analyzed for CD107a expression by flow cytometry and IFNγ production was determined as previously described (Alvarez *et al*, 2020b). In other experiments, the tumor component (CD45^−^) was stained with anti‐calreticulin (Abcam) or active anti‐caspase‐3 (BD Bioscience) following permeabilitation per manufacturer's instructions.

In all conditions, cells were first stained for the live/death fixable maleimida marker PF840 (PromoFluor) per manufacture's instructions. For a detailed description of the mAbs used, refer to Appendix Table S1.

### Cytotoxicity experiments

Purified mouse activated NK cells were incubated with/without Fc‐Block to evaluate ADCC‐mediated cytotoxicity followed by incubation of 10 μg/ml of anti‐mouse NKG2A/C/E (clone 20d5) (Vance *et al*, 1998) or rIgG2aκ (eBioscience™, San Diego, CA) for 30 min at 4°C. Concurrently, C57BL/6‐derived tumor cells were incubated with 5 μg/ml anti‐mouse Qa‐1^b^ or mIgG1 (BioXcell, Lebanon, NH) for 30 min at 4°C and then stained with 2.5 μM of CellTrace™ CFSE per manufacturer's instructions (Invitrogen, Waltham, MA). NK cells and CFSE‐labeled tumor cells were then co‐cultured at the indicated effector: target (E:T) rations for 4 h at 37°C. The same culture conditions were employed when the Cr^51^‐release assay was used to analyze NK cell cytotoxicity. In these experiments, Qa‐1^b^ pre‐incubated tumor cells were cultured with 2 mCi/ml for 1 h at 37°C followed by two washes with RPMI complete media. Next, NK cells and Cr^51^‐labeled tumor cells were then co‐cultured at the indicated effector: target (E:T) ratios for 4 h at 37°C with or without the presence of the indicated mAb. The percentage of tumor lysis was calculated as previously described (Alvarez *et al*, 2016).

For experiments using human NK cells, 14‐day cultured rhIL‐2 expanded NK cells were co‐culture with Cr^51^‐labeled CAL‐27 HLA‐E^low^ or CAL‐27 HLA‐E^high^ at different E:T ratios following the culture conditions described by Andre and collaborators (Andre *et al*, 2018).

### Mouse tumor models

4 × 10^5^ MC38, B16.OVA or CT26 cells were subcutaneously injected in the right flank of C57BL/6 (MC38 or B16.OVA) or BALB/c mice (CT26). When tumors reached 30–40 mm^3^ approximately at day six post‐inoculation, mice were randomized and treated intratumorally with 50 μl of 30 μg anti‐mouse Qa‐1^b^ mAb or mouse IgG1 (BioXcell). The following day, mice received intratumoral injections of 100 or 30 μg anti‐mouse NKG2A/C/E mAb or rat IgG (BioXcell) along with 2 × 10^6^ of syngeneic or allogeneic activated donor NK cells that had been expanded *in vivo* from C57BL/6 or BALB/c‐derived Rag1^−/−^ mice previously treated with hydrodynamic injections in the tail vein of a plasmid cDNA encompassing Sushi‐IL15‐Apo (Ochoa *et al*, 2013; Alvarez *et al*, 2020a) as corresponded. In some experiments, *ex vivo* expanded NK cells were pre‐incubated with 30 μg/50 μl of anti‐NKG2A or rIgG for 30 min at 4°C and cells were washed twice with PBS prior to intratumoral injection of the rIgG or anti‐NKG2A pre‐incubated NK cells.

For selective depletion studies, mice received intraperitoneal (ip) injections of 100 μg anti‐CD8β (clone Lyt 3,2, BioXcell), anti‐CD4 (clone GK1.5, BioXcell) or anti‐NK1.1 (clone PK136, BioXcell) mAbs on days five and seven post‐tumor inoculation followed by weekly ip injections until the end of the experiment to deplete endogenous CD8, CD4, and NK cells, respectively, without altering the CD8α^+^ DC compartment. Control mice received ip injections of rIgG. To evaluate the role of cDC1, BATF3^−/−^ mice or their corresponding counterparts were used.

In some experiments, 100 μg anti‐PD‐1 mAb (clone RMP1‐14, BioXcell) or isotype control rIgG were given intraperitoneally on days seven, nine and 11 after tumor inoculation. For rechallenge studies, tumor‐free surviving mice that were treated with anti‐PD‐1 and NK + anti‐NKG2A/Qa‐1^b^ intratumoral therapy were injected subcutaneously with 2 × 10^5^ MC38 tumor cells on day 45–60 after the first tumor MC38 inoculation. As control for tumor growth, naïve resting C57BL/6 mice were also challenge with MC38. For bilateral tumor mouse models, mice were sc injected with 4 × 10^5^ and 2 × 10^5^ MC38 tumor cells in the right and left flank, respectively, but only the right flank was treated. Treated and untreated tumors were measured twice a week with calipers and the volume was calculated (length × width^2^/2). Additionally, mice were monitored for survival and euthanized when any tumor reached a diameter of 15 mm or mice displayed signs of distress per ECAE guidelines. The tumor size of sacrificed mice is not included for the calculation of the average tumor size once they have been sacrificed or found death.

### Analysis of exogenous NK cells

CD45.1 C57BL/6 mice were used as NK cell donors to distinguish between exogenous and endogenous (CD45.2) NK cells in tumor‐bearing CD45.2 C57BL/6 mice. In these experiments, NK donor mice were intraperitoneally injected with anti‐100 μg anti‐CD8β (clone Lyt3.2, BioXcell) and anti‐CD4 (clone GK1.5, BioXcell) mAbs prior to hydrodynamic injections with plasmid cDNA encompassing Sushi‐IL15‐Apo on days −2 and 0. Purified CD45.1 NK cells were then intratumorally injected into MC38 tumor‐bearing CD45.2 C57BL/6 mice with or without anti‐NKG2A/Qa‐1^b^ injections. Tumors were collected 4 days post NK cell treatment. Single‐cell suspensions were stained with anti‐mouse CD45, TCRβ, CD19, NK1.1 and CD45.2 mAbs and the percentage of exogenous (CD45.2^−^) and endogenous (CD45.2^+^) NK cells was determined by flow cytometry. For a detailed description of the mAbs used, refer to Appendix Table S1.

### Human materials

Peripheral blood mononuclear cells (PBMCs) were obtained from healthy donors (female and male) following written, signed, and dated informed consent according to a protocol approved by the institutional ethics committee following the principles set out in the WMA Declaration of Helsinki and the Department of Health and Human Services Belmont Report.

### Human NK cell isolation and culture

Total lymphocytes were isolated from PBMCs by Cytiva Ficoll‐paque™ Plus (Sigma‐Aldrich) density gradient centrifugation, and then NK cells were purified using the EasySep™ Human NK cell Isolation Kit (StemCell Technology) by immunomagnetic negative selection following the manufacturer's instructions. For NK‐cell expansion, NK cells were co‐cultured for 14‐days with autologous irradiated (20Gy) NK cell‐depleted lymphocytes at a 20:1 feeder:NK cell ratio. NK cells were grown in complete NK cell media additionally supplemented with 8% human AB serum and 1 μ/ml phytohemagglutinin. Half of the NK cell media was replaced every 3–4 days, and 10 ng/ml of anti‐CD3 mAb (functional grade OKT3, Miltenyi Biotec) was added to the media the first 4 days. In addition, 500 IU/ml rhIL‐2 was freshly added to the media each time. NK cell purity and expansion were evaluated by flow cytometry.

### HLA‐E plasmid construct and transfection

A plasmid coding for the signal peptide of the HLA‐G molecule and the HLA‐E sequence (Appendix Table S2) was generated from insertions at the site SpeI/BsiWI into the pT4/HB plasmid (#108352 Addgene) by Genescript (Piscataway, NY). This plasmid was designed to have the insert flanked by tandem repeats recognized by sleeping beauty transposase. The plasmid coding for transposase (SB13) was kindly provided by Dr. Lujambio and generated as previously described (Ruiz de Galarreta *et al*, 2019).

CAL‐27 cells were maintained overnight to 80 percent confluence and transfected with the GpHLA‐E containing plasmid using lipofectamine 2000 (Invitrogen) following the manufacturer's protocols. Tumor cells were then stained with anti‐human HLA‐E and HLA‐E^+^ cells were single‐cell sorted with a MoFlo Astrios EQ cell sorter (Beckmann Coulter). Single‐cell clones were expanded in culture using complete RPMI media.

### Xenograft mouse model

Immunodeficient BALB/c Rag2^−/−^IL2Rγ^−/−^ mouse were inoculated subcutaneously with 5 × 10^5^ Cal‐27 HLA‐E^high^ tumor cells in the right flank. Seven days post‐tumor inoculation when tumors reach 25–40 mm^3^, mice received intratumoral injections of 8 millions of activated human NK cells and/or 50 μg of monalizumab. Control mice were treated with PBS. In some experiments, mice were inoculated with Cal‐27 HLA‐E^high^ and Cal‐27 HLA‐E^low^ tumor cells in the right and left flack, respectively, and both tumors were equally treated. Tumors were measured with a caliper twice a week, and mice were sacrificed when tumors reach 15 mm of diameter or mice display any signal of distress per ECAE guidelines.

### Statistical analysis

For *in vivo* experiments, mice were randomized in an unbiased fashion. Researchers were not blinded during the experiments. Samples sizes were estimated based on power analysis of data from our previous studies to achieve a statistical power of 80% and *P*‐value of 0.05 in efficacy between the combination and either individual treatment arm. Each experiment was performed with 6–5 mice per group. The Student's two‐tailed *t*‐test, one‐way ANOVA (Bonferroni post‐test analysis), two‐way ANOVA (Bonferroni post‐test analysis), or log‐rank test was used when appropriate for statistical comparisons (Graphpad Prism 6, La Jolla, CA). Longitudinal data were fitted to the third‐order polynomial equation and compared with an extra sum‐of‐squares *F* test. Log‐rank test *P*‐values were considered statistically significant when *P* < 0.05.

## Author contributions

**Ignacio Melero:** Conceptualization; resources; formal analysis; supervision; investigation; methodology; writing – original draft; project administration; writing – review and editing. **Maria C Ochoa:** Data curation; formal analysis; methodology; writing – review and editing. **Carmen Molina:** Validation; methodology; writing – review and editing. **Sandra Sanchez‐Gregorio:** Methodology. **Saray Garasa:** Data curation; methodology. **Carlos Luri‐Rey:** Visualization; writing – review and editing. **Sandra Hervás‐Stubbs:** Methodology; writing – review and editing. **Noelia Casares:** Data curation; investigation; methodology; writing – review and editing. **Edurne Elizalde:** Investigation; methodology. **Gabriel Gomis:** Methodology; writing – review and editing. **Assunta Cirella:** Methodology; writing – review and editing. **Pedro Berraondo:** Conceptualization; supervision; methodology; writing – review and editing. **Alvaro Teijeira:** Data curation; visualization; methodology; writing – review and editing. **Maite Alvarez:** Conceptualization; data curation; formal analysis; supervision; investigation; methodology; writing – original draft; writing – review and editing.

## Disclosure and competing interests statement

CM, SS‐G, SG, SH‐S, NC, EE, GG, AC, PB and AT declare no competing interests. MCO reports receiving a commercial research grant from AstraZeneca. IM reports receiving commercial research grants from AstraZeneca, BMS, Highlight Therapeutics, Alligator, Pfizer Genmab and Roche; has received speakers bureau honoraria from MSD; and is a consultant or advisory board member for BMS, Roche, AstraZeneca, Genmab, Pharmamar, F‐Star, Bioncotech, Bayer, Numab, Pieris, Gossamer, Alligator and Merck Serono. MA reports receiving commercial grants from Pharmamar and Highlight Therapeutics.

## Supporting information



Appendix S1

Expanded View Figures PDF

PDF+

Source Data for Figure 1

Source Data for Figure 2

Source Data for Figure 3

Source Data for Figure 4

Source Data for Figure 5

Source Data for Figure 6

Source Data for Figure 7

## Data Availability

This study includes no data deposited in external repositories.
