# Supplementary material for: Intratumoral co‐injection of NK cells and NKG2A‐neutralizing monoclonal antibodies
Source: EMBO Mol Med. 2023 Oct 2;15(11):e17804. doi: 10.15252/emmm.202317804 (PMC10630884; doi:10.15252/emmm.202317804)

TABLE OF CONTENT

	Page
1. Appendix Table S1	2
2. Appendix Table S2	3
3. Appendix Figure Legends S1	4
4. Appendix Figure Legends S2	4
5. Appendix Figure Legends S3	4
6. Appendix Figure Legends S4	5
7. Appendix Figure Legends S5	5
8. Appendix Figure Legends S6	5
9. Appendix Figure S1	7
10. Appendix Figure S2	8
11. Appendix Figure S3	9
12. Appendix Figure S4	10
13. Appendix Figure S5	11
14. Appendix Figure S6	12

Appendix Table S1. Antibodies for flow cytometry. The table lists all antibodies used in this study, including clone, provider and catalogue number.

Antibody	Clone	Provider	Catalogue	Dilution
**CD11b**	M1/70	BioLegend	101259	1:400
**CD11c**	N418	BioLegend	117334	1:200
**CD16**	3G8	BioLegend	302006	1:200
**CD19**	6D5	BioLegend	115541	1:200
**CD25**	PC61	BioLegend	102012	1:200
**CD3**	UCHT1	BioLegend	300416	1:200
**CD4**	GK1.5	BD Bioscience	612952	1:200
**CD44**	IM7	BioLegend	103006	1:100
**CD45**	30.F11	BioLegend	103138	1:200
**CD45.2**	104	BioLegend	109820	1:200
**CD49a**	HMα1	BioLegend	142603	1:100
**CD56**	HCD56	BioLegend	318348	1:100
**CD62L**	MEL-14	BioLegend	104448	1:200
**CD8**	53-6.7	BD Bioscience	563795	1:200
**CXCR6**	DANID2	Thermo Fisher Scientific	46-9186-82	1:200
**F4/80**	BM8	BioLegend	123114	1:400
**Foxp3**	MF-14	BioLegend	126419	1:200
**Gp70**		Proimmune	KSPWFTTL	1:50
**HLA-E**	3D12	BioLegend	342610	1:50
**Ki67**	16A8	BioLegend	652420	1:800
**Ly6C**	HK1.4	BioLegend	128006	1:400
**Ly6G**	1A8	BioLegend	127633	1:400
**MHCII**	M5/114.15.2	Thermo Fisher Scientific	47-5321-82	1:400
**NK1.1**	PK136	BioLegend	108148	1:160
**NKG2A**	16A11	BioLegend	142809	1:100
**NKG2A**	375113	BioLegend	375113	1:100
**NKG2C**		R&D Systems	FAB1382R	1:100
**Qa-1b**	6A8.6F10.1A6	BD Bioscience	559829	1:100
**TCRb**	H57-597	BioLegend	109249	1.200

Appendix Table S2. Sequence coding for the signal peptide of the HLA-G molecule and the HLA-E.

CGTACGTTACAAGCTGTGAGACTCAGACCCCTGGGCACTGTCGCTCCACTCAGCCTTAGAGTAGCTCCCTCCTT

TTCCACCTGAGCTCTTCTTCCTCCATATCACAGCAGCAACCACAGCTCCAGAGACCACAGATCCAAGGAGAACC

AGGCCAGCAATGATGCCCACGATGGGGATGGTGGGCTGGGAAGCCGGCTTCCATCTCAGGGTGACGGGCTC

GGGTAGCCCCTCATGCTGCACATGGCACGTGTATCTCTGCTCCTCTCCAGAAGGCACCACCACAGCTGCCCACT

TCTGGAAGGTTCCATCCCCTGCAGGCCTGGTCTCCACGAGCTCCGTGTCCTGGGTATGGCCCTCCCCATCCTGC

TGCCAGGTCAGTGTGATCTCCGCAGGGTAGAAGCCCAGGGCCCAGCACCTCAGGGTGGCCTCATGGTCAGAG

ATGGGGTGGTGAGTCACGTGTGTCTTTGGGGGCTCCAGGTGAAGCAGCGTCTCCTTCCCCTTCTCCAGGTATT

TGTGGAGCCACTCCACGCATGTGTCTTCCAGGTAGGCTCTCTGGTGCTCCGCCTCAGAGGCATCATTTGACTTT

TGCTCGGAGATCTGAGCCGCCGTGTCCACCGCGGTCCAGGAGCGCAGGTCCTCATTCAGGGTGAGATAATCC

TTGCCGTCGTAGGCGAACTGTTCATACCCGCGGAGGAAGCGCCCGTCGGGCCCCAGCTCGCAGCCATGCATC

CACTGCAGGGTGTGAGACCCGGCCTCGCTCTGATTGTAGTAGCCGCGCAGCGTCCGCAGATTCACTCGGAAA

ATCTGTGCGGTGTCCCTGGCGCTCCGTGTCTCCCGGTCCCAATACTCTGACCCCTCCTGCTCCATCCACGGCGC

CCGCGGCACCATCCTCGGACTCGCGGCGTCGTTGTCGAAGCGCACGAACTGGGTGTCGTCCACGTAGCCCAC

AGAGATGAAGCGGGGCTCCCCGCGGCCGGGCCGGGACACGGAAGTGTGGAAATACTTCAAGGAGTGGGAG

CCcgcccaggtctcggtcagggtcagggcccccgagagcagcaggaagagggttcggggcgccatgaccaccatggtggcggcACTAGT

APPENDIX FIGURE LEGENDS

Appendix Figure S1. Distribution of NKG2A and NKG2C expression on NK cells. (A-B) Representative dot-plots (A) and total percentage (B) of NKG2A and NKG2C expression on gated NK cells ($CD45^{+}$ $TCR\beta^{-}NK1.1^{+}$) after control or plasmid hydrodynamic injection of ApoAI-IL-15-Sushi. Data are representative of two independent experiments with four mice per group (mean ± SEM). One-Way ANOVA was used to assess significance (****p<0.0001).

Appendix Figure S2. Blockade of the NKG2A/Qa-1^{b} interaction enhances anti-tumor cytotoxicity of IL-15 activated mouse NK cells. (**A-B**) Representative histograms (A) and total percentage (B) of Qa-1b expression are shown for control and IFNγ-stimulated tumor cell lines corresponding to MC38 (upper panel) and B16.OVA (lower panel). (**C**) The percentage of tumor lysis mediated by NK cells against MC38 (upper panel) and B16.OVA (lower panel) is shown for each treatment condition. Data are representative of two independent experiments performed in triplicate (mean ± SEM). Student T-test analysis (B) or a two-way ANOVA analysis (C) were used to assess statistical significance. In C, significant differences are displayed for comparisons of each group with the NK + anti-NKG2A/Qa-1^{b} group (****p<0.0001).

Appendix Figure S3. The intratumoral injections of NK cells and anti-NKG2A/Qa1^{b} does not increase the functional capabilities of tumor infiltrating CD8 T cells. (A) Schematic representation of the doses and treatment regimens followed. Control mice and NK cell-treated mice intratumorally received mIgG1 and rIgG at the same dose as a control. **(B)** Representative dot-plot images of CD107a expression on gated intratumoral CD8 T cells ($CD45^{+}CD19^{-}TCR\beta^{+}NK1.1^{-}CD4^{-}CD8^{+}$). **(C)** Total percentage of CD107a on CD8 T cells from collected tumors at day 7 post-NK cell intratumoral injection. **(D)** Representative images of induced intracellular IFNγ expression by CD8 T cells. **(E)** Total percentage of IFNγ producing CD8 T cells is shown. Data represents an experiment with five mice per group (mean ± SEM). One-Way Anova were used to assess significance. Significant differences are displayed for comparisons of each group with the NK + anti-NKG2A/Qa-1^{b} group.

Appendix Figure S4. Exogenous NK cells disappear shortly after intratumoral injections. (A) The total number (left panel) and percentage (right panel) of total NK cells within the TME is shown 14 days post-tumor inoculation. **(B)** The percentage of Ki67 is shown for gated NK cells. IL-15 activated $CD45.1^+$ C57BL/6-derived NK cells were injected into $CD45.2^+$ C57BL/6 tumor-bearing mice and the presence of exogenous ($CD45.2^-$) and endogenous ($CD45.2^+$) NK cells was evaluated by flow cytometry four days after NK-cell transfer (11 days post-tumor inoculation). **(C)** Schematic representation of the regimen followed. **(D)** Representative dot-plots (left panel) or total percentages of CD45.2 expression on FACS-gated NK cells are shown for each treatment condition. Data represents one experiment with five-six mice per group (mean ± SEM). A One-Way ANOVA was used to determine statistical significance.

Appendix Figure S5. The blockade of NKG2A only on NK cells is enough to lead the anti-tumor response caused by intratumoral injections of NK cells + anti-NKG2A/$Qa1^b$. Prior to intratumoral injections, ex vivo expanded NK cells were incubated with anti-NKG2A (anti-NKG2A NK) or rIgG (rIgG NK) for 30 min at 4ºC and then washed twice with PBS. **(A)** Schematic representation of the treatment regimens followed. Control and NK cell-treated mice were intratumorally injected together with isotype-matched controls mIgG1 and rIgG when indicated. **(B-C)** The average tumor volume (mm^3) to follow in vivo tumor progression (B) and the percentage of survival (C) are shown over time for each treatment condition. Data represents an experiment with six mice per group (mean ± SEM). In B, data were fitted to a third-order polynomial and compared using Extra sum-of-squares F test. In C, Log-rank tests were used to assess significance. Significant differences are displayed for comparisons of each group with the rIgG NK + anti-NKG2A/$Qa\text{-}1^b$ group or anti-NKG2A NK + anti-$Qa1^b$ (****p<0.0001).

Appendix Figure S6. Calreticulin exposure on malignant cells upon treatment indicates immunogenic cell death. MC38-derived tumors were collected seven days post-NK and anti-NKG2A injections and tumors were analyzed for surface calreticulin and intracellular active caspase-3 by flow cytometry. **(A)** Schematic representation of the dose regimen followed. **(B)** Representative dot-plot images showing the expression of calreticulin on viable tumor cells ($CD45^-PF840^-$). **(C)** Total

percentage of calreticulin exposure on tumor cells gated as $CD45^{-}PF840^{-}$ in cell suspensions from MC38 excised tumors. **(D)** Representative dot-plot images of caspase-3 on $CD45^{-}PF840^{-}$ tumor cells. **(E)** Total percentage of caspase-3 on tumor cells. Data represents an experiment with five mice per group (mean ± SEM). One-Way Anova tests were used to assess significance. Significant differences are displayed for comparisons of each group with the NK + anti-NKG2A/Qa-1^{b} group (****$p<0.0001$)

Appendix Figure S1

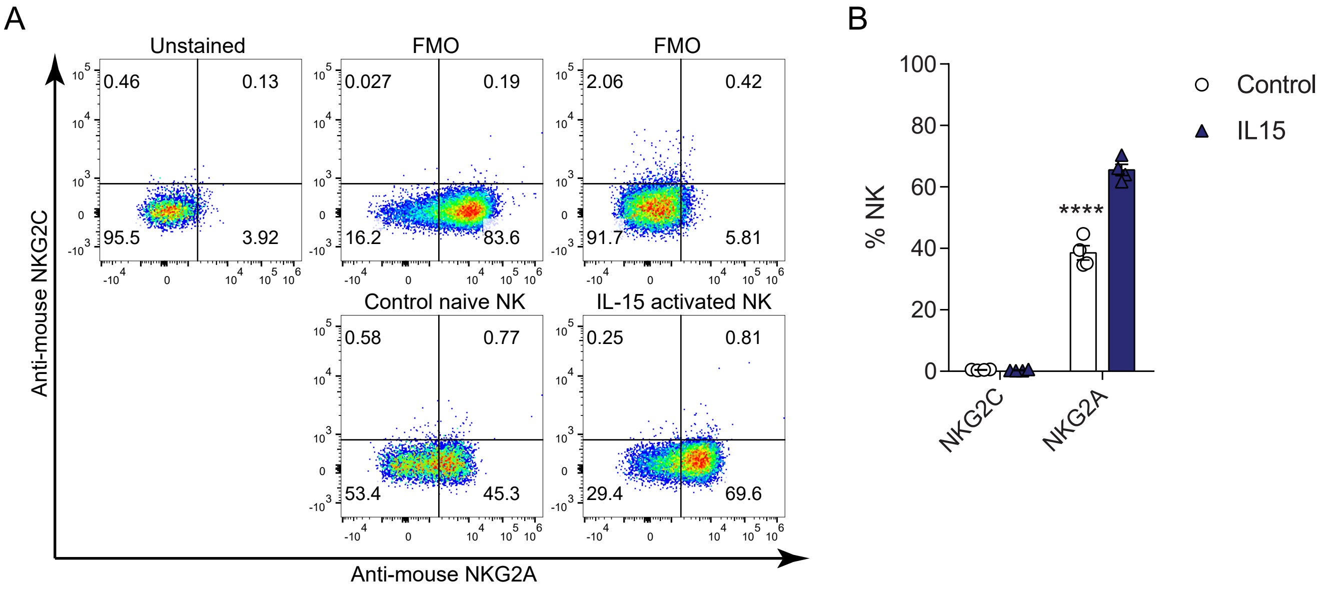

Appendix Figure S2

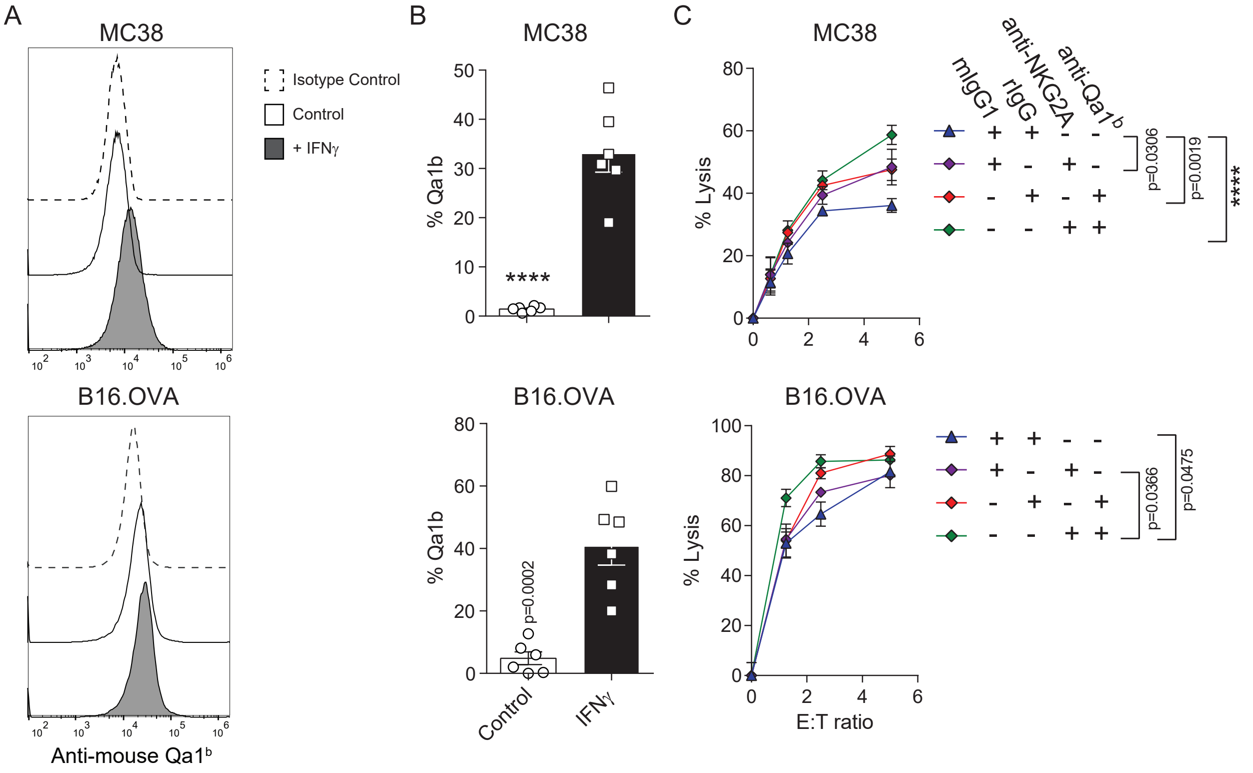

Appendix Figure S3

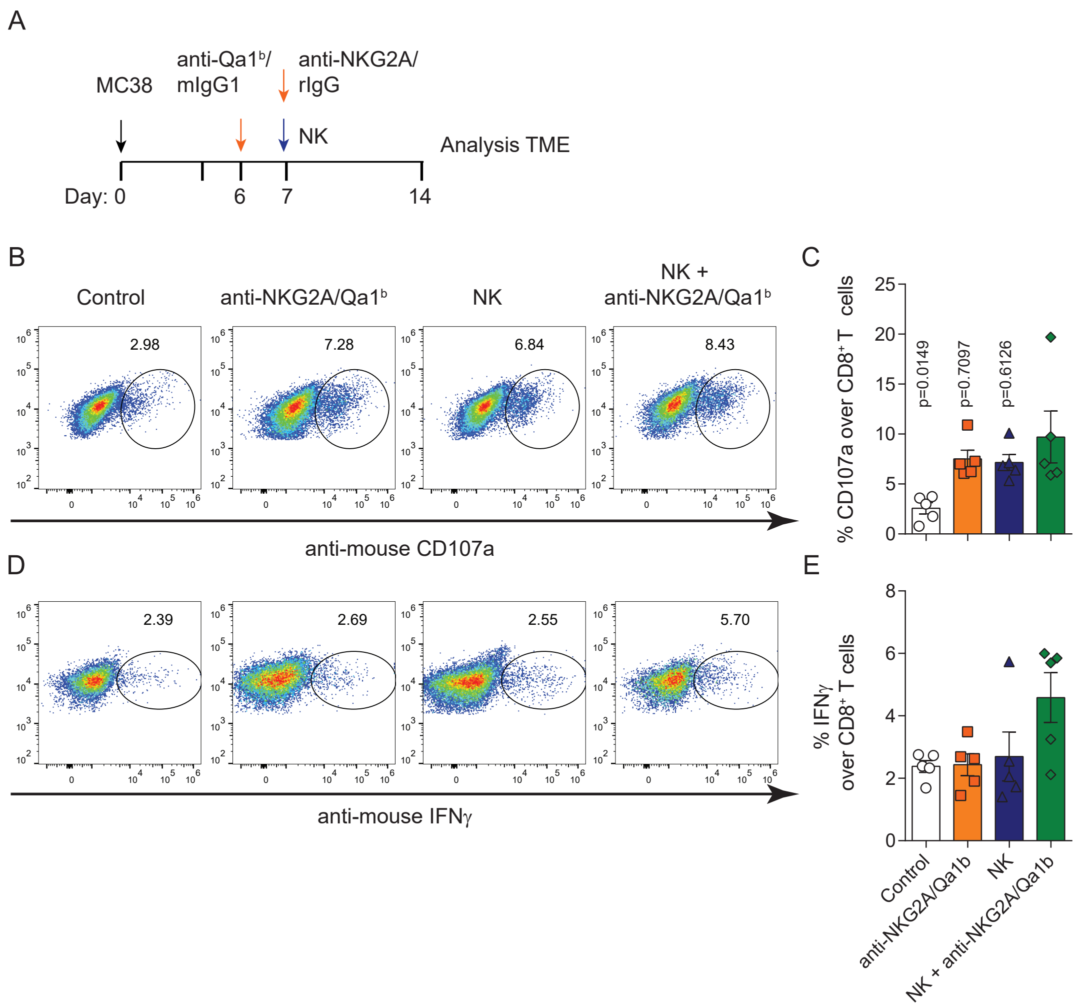

Appendix Figure S4

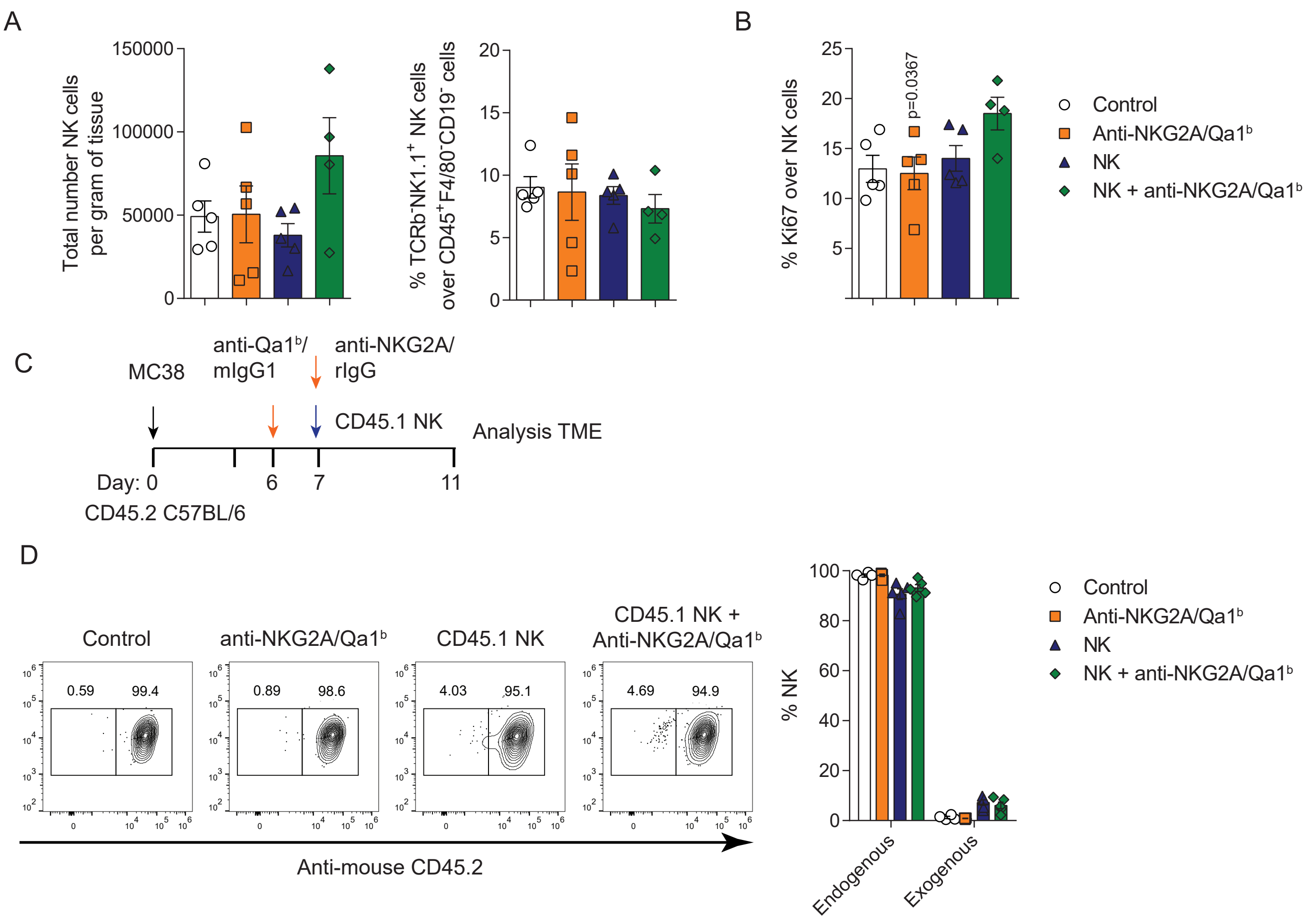

Appendix Figure S5

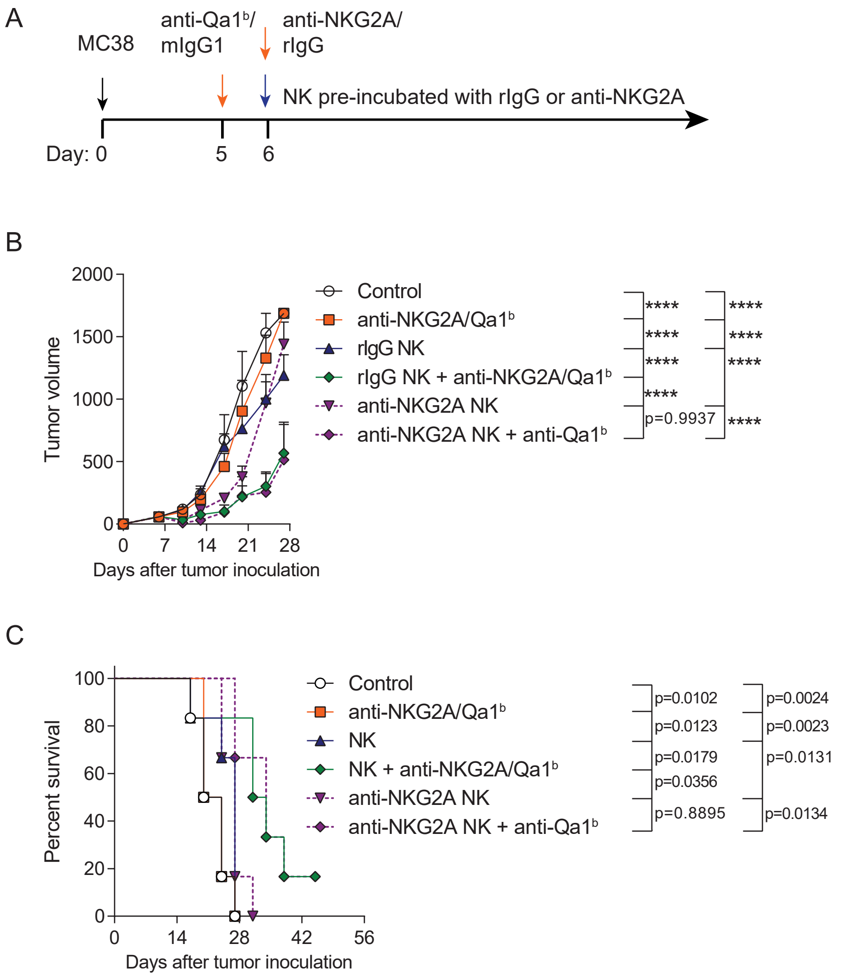

Appendix Figure S6

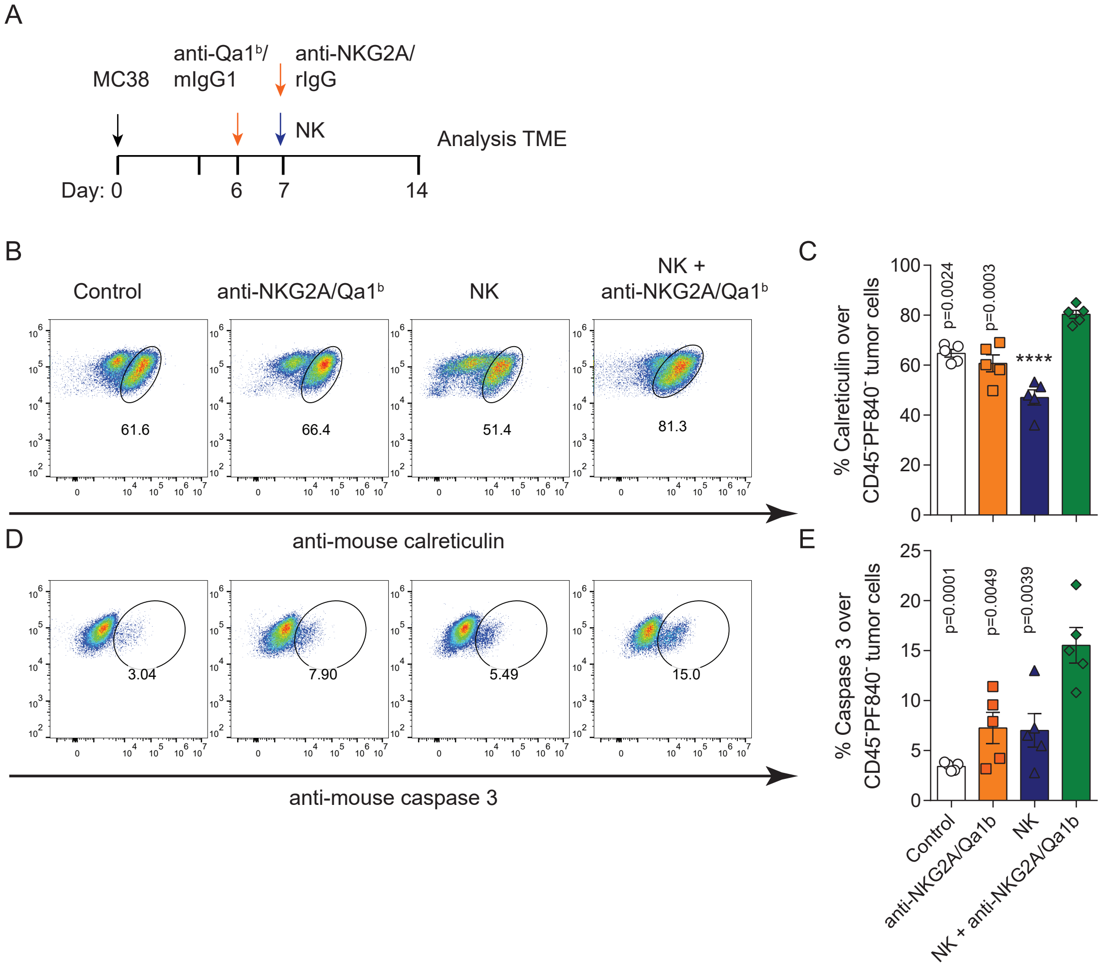

Supplement: Supplementary file 1 — Appendix S1 [file EMMM-15-e17804-s006.pdf]
