# Supplementary material for: Intratumoral co‐injection of NK cells and NKG2A‐neutralizing monoclonal antibodies
Source: EMBO Mol Med. 2023 Oct 2;15(11):e17804. doi: 10.15252/emmm.202317804 (PMC10630884; doi:10.15252/emmm.202317804)
Supplement: Supplementary file 2 — Expanded View Figures PDF [file EMMM-15-e17804-s007.pdf]

Expanded View Figures

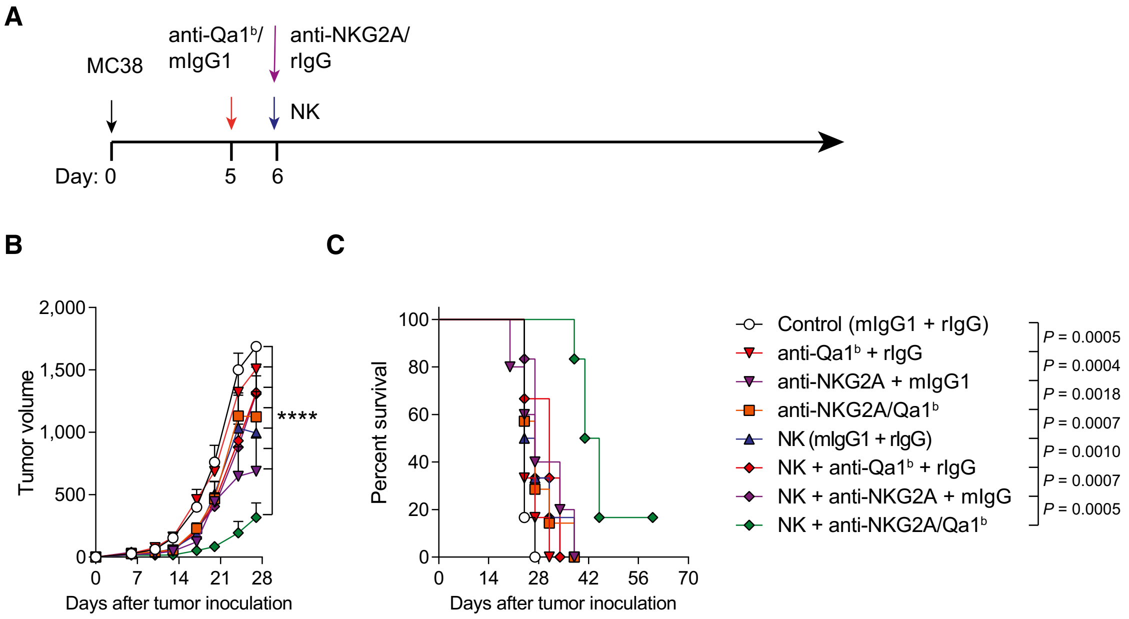

Figure EV1. The dual blockade of Qa-1b and NKG2A within the tumor site better controls tumor growth when combined with activated NK cell intratumoral administration.

A–C (A) Schematic representation of the regimens followed. mIgG1 and/or rIgG isotype controls were injected when corresponding. (B) The tumor volume (mm^3) progression is shown over time for each treatment condition (mean ± SEM). (C) The percentage of survival of the indicated groups is shown.

Data information: Data are representative of three independent experiments with six mice per group (mean ± SEM). In (B), data were fitted to a third-order polynomial and compared using extra sum-of-squares *F* test. In (D), log-rank tests were used to assess significance. Significant differences are displayed for comparisons of each group with the NK + anti-NKG2A/Qa-1^b group (*****P* < 0.0001).

© 2023 The Authors

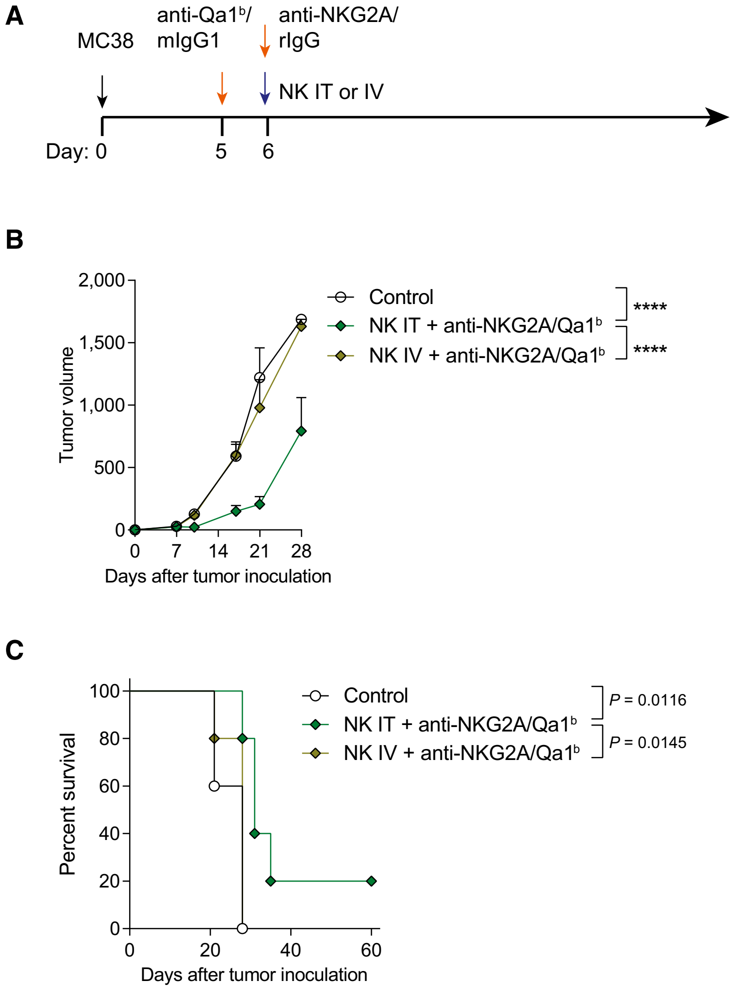

Figure EV2. The synergy between anti-NKG2A/Qa1^b and NK cells is only detectable when NK cells are given intratumorally, but not intravenously.

A–C (A) Schematic representation of the tumor treatment regimen followed in which mice received intratumoral (IT) or intravenous (IV) treatment. Control mice were treated with control mIgG1 and rIgG when indicated. (B) Tumor volume (mm^3) is shown for each treatment condition. (C) The percentage of survival is shown.

Data information: Data represent an experiment with five mice per group (mean ± SEM). In (B), data were fitted to a third-order polynomial and compared using extra sum-of-squares *F* test. In (C), log-rank tests were used to assess significance (****$P < 0.0001$).

 © 2023 The Authors

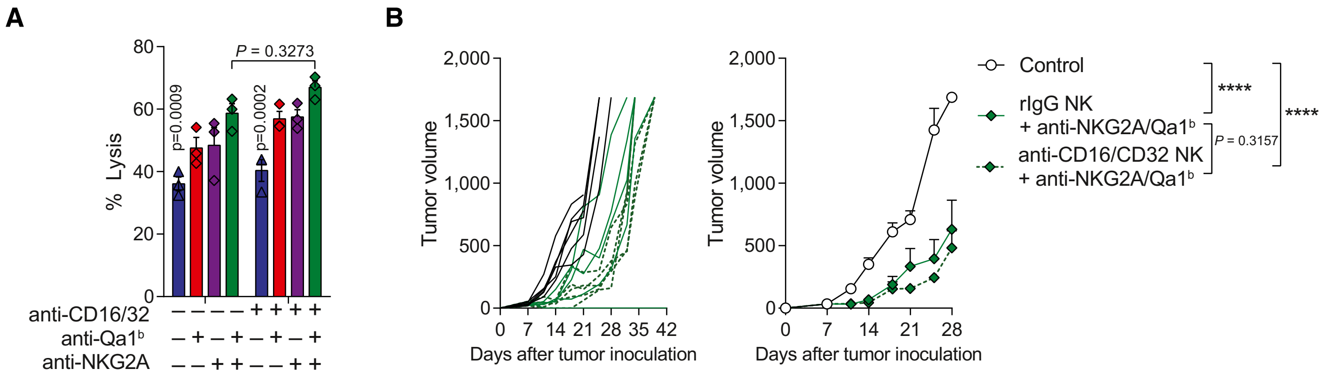

Figure EV3. Anti-Qa-1^b-mediated ADCC does not play a role in the NK and anti-NKG2A/Qa-1^b immunotherapeutic efficacy.

A, B Activated NK cells were incubated with anti-CD16/CD32 or control isotype prior to evaluating their cytotoxicity *in vitro* in a standard 4 h CFSE-based killing assay. (A) The percentage of tumor lysis is shown for each condition at 5:1 E:T ratio. Isotype control or anti-CD16/CD32 pretreated NK cells were intratumorally injected following the regimen schedule described in Fig 2A. (B) Tumor growth is shown over time for each individual mice (left panel) or treatment group (right panel).

Data information: Data are representative of two independent experiments performed in triplicate (A) or with six mice per group (B) (mean ± SEM). Two-way ANOVA (A) or extra sum-of-squares *F* test of the data fitted to a third-order polynomial (B) were used to assess significance (*****P* < 0.0001).

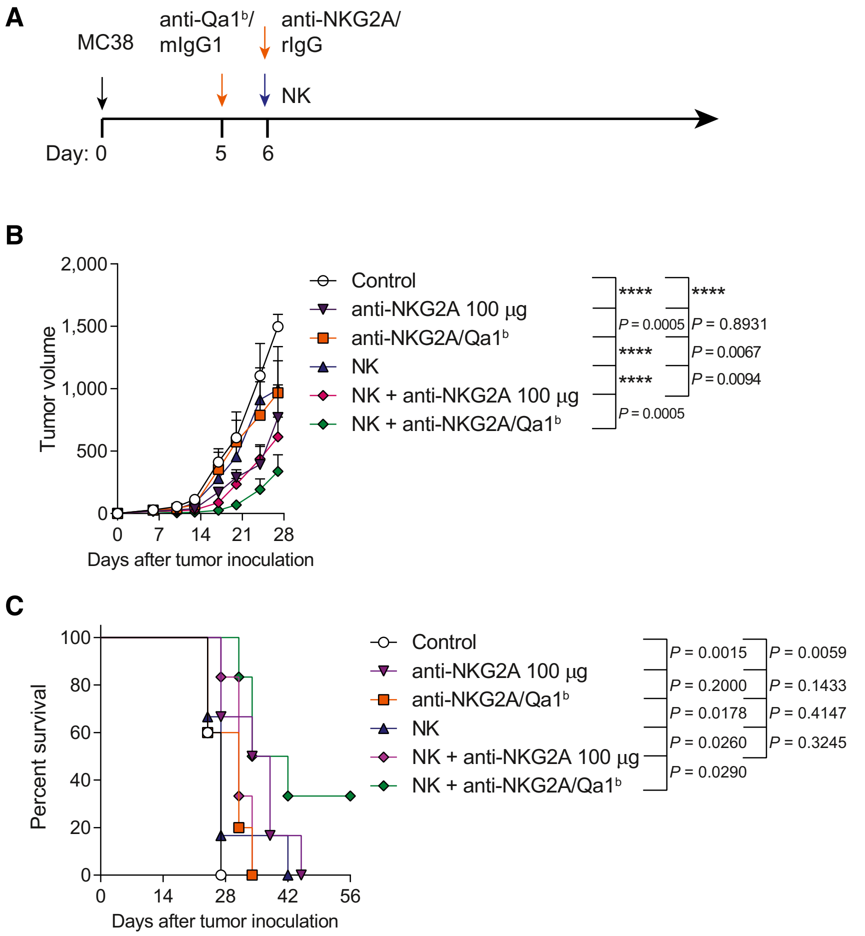

Figure EV4. The intratumoral injections of low dose anti-NKG2A and anti-Qa1^b are more effective than a high dose of anti-NKG2A.

A Schematic representation of the dose regimen followed. Control mice and NK cell-treated mice intratumorally received mIgG1 and rIgG at the same dose as a control.

B, C The average tumor volume (mm^3) for average tumor progression (B) and the percentage of survival (C) are shown over time.

Data information: Data represent an experiment with 5–6 mice per group (mean ± SEM). In (B), data were fitted to a third-order polynomial and compared using extra sum-of-squares *F* test. In (C), log-rank tests were used to assess significance. Significant differences are displayed for comparisons of each group with the NK + anti-NKG2A/Qa-1^b group or NK + anti-NKG2A 100 µg (*****P* < 0.0001).

© 2023 The Authors

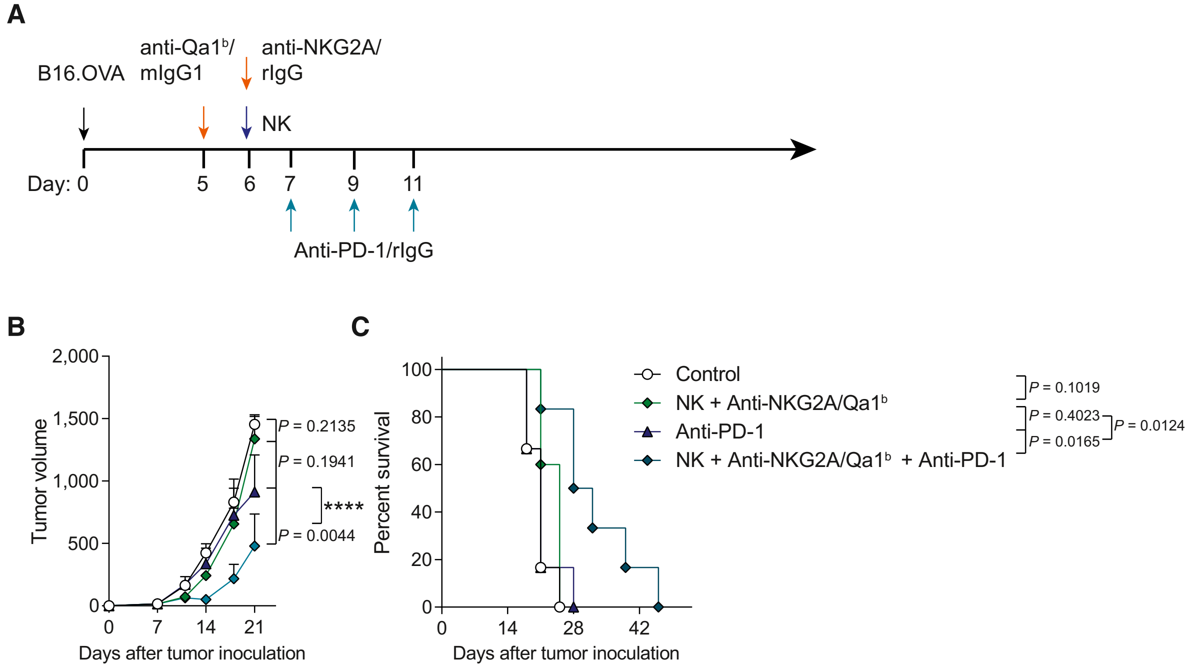

Figure EV5. Anti-PD-1 therapy synergizes with intratumoral NK and anti-NKG2A/Qa-1^b therapy to better control the growth of B16.OVA melanoma.

A–C (A) Schematic representation of the regimes followed. Control mice and NK cell-treated mice received mIgG1 and rIgG. (B) The means of tumor size (mm^3) is shown over time for each treatment condition. (C) The percentage of survival as shown in a Kaplan–Meier graph.

Data information: Data are representative of two independent experiments with six mice per group. Extra sum-of-squares *F* test of the data fitted to a third-order polynomial (B) or log-rank tests were used to assess significance. Significant differences are displayed for comparisons of each group with the NK + anti-NKG2A/Qa-1^b group or the NK + anti-NKG2A/Qa-1^b + anti-PD-1 group (****$P < 0.0001$).

 © 2023 The Authors
