# Supplementary material for: Intratumoral co‐injection of NK cells and NKG2A‐neutralizing monoclonal antibodies
Source: EMBO Mol Med. 2023 Oct 2;15(11):e17804. doi: 10.15252/emmm.202317804 (PMC10630884; doi:10.15252/emmm.202317804)
Supplement: Supplementary file 11 [file EMMM-15-e17804-s003.pdf]

EMBO Molecular Medicine

Intratumoral co-injection of NK cells and NKG2A-neutralizing monoclonal antibodies

Ignacio Melero, María del Carmen Ochoa, Carmen Molina, Sandra Sanchez-Gregorio, Saray Garasa, Carlos Luri-Rey, Sandra Hervás-Stubbs, Noelia Casares, Edurne Elizalde, Gabriel Gomis, Assunta Cirella, Pedro Berraondo, Alvaro Teijeira, and Maite Alvarez
DOI: 10.15252/emmm.202317804

Corresponding authors: Ignacio Melero (imelero@unav.es) , Maite Alvarez (malvarezr@unav.es)

Review Timeline:

Submission Date:	4th Apr 23
Editorial Decision:	8th May 23
Revision Received:	28th Jul 23
Editorial Decision:	12th Aug 23
Revision Received:	18th Aug 23
Editorial Decision:	1st Sep 23
Revision Received:	4th Sep 23
Accepted:	7th Sep 23

Editor: Lise Roth

Transaction Report:

(Note: With the exception of the correction of typographical or spelling errors that could be a source of ambiguity, letters and reports are not edited. Depending on transfer agreements, referee reports obtained elsewhere may or may not be included in this compilation. Referee reports are anonymous unless the Referee chooses to sign their reports.)

8th May 2023

Dear Dr. Melero,

Thank you for the submission of your manuscript to EMBO Molecular Medicine, and please accept my apologies for the delay in getting back to you, as we were expecting an additional report. Unfortunately, reviewer #3 has not gotten back to us despite several chasers, and since the other 2 reviewers provide similar recommendations, we decided to make a decision with the reports at hand in order not to delay the process further. Should referee #3 provide a report, we will send it to you with the understanding that we will not ask for extensive experiments in addition to the ones already included here.
As you will see below, the reviewers raise substantial concerns on your work, which unfortunately preclude its publication in EMBO Molecular Medicine in its current form.

If you feel you can satisfactorily address the points listed by the referees, you may wish to submit a revised version of your manuscript. In particular, it will be important to highlight the novel aspects of your manuscript in light of previous publications. Please attach a covering letter giving details of the way in which you have handled each of the points raised by the referees. A revised manuscript will once again be subject to review and we cannot guarantee at this stage that the eventual outcome will be favorable. EMBO Molecular Medicine encourages a single round of revision only and therefore, acceptance or rejection of the manuscript will depend on the completeness of your responses included in the next, final version of the manuscript. For this reason, and to save you from any frustrations in the end, I would strongly advise against returning an incomplete revision.

If you would like to discuss further the points raised by the referees, I am available to do so via email or video. Let me know if you are interested in this option.

Revised manuscripts should be submitted within three months of a request for revision; they will otherwise be treated as new submissions, except under exceptional circumstances in which a short extension is obtained from the editor.

When submitting your revised manuscript, please carefully review the instructions that follow below. We perform an initial quality control of all revised manuscripts before re-review; failure to include requested items will delay the evaluation of your revision.

We require:

1) A .docx formatted version of the manuscript text (including legends for main figures, EV figures and tables). Please make sure that the changes are highlighted to be clearly visible.

2) Individual production quality figure files as .eps, .tif, .jpg (one file per figure). For guidance, download the 'Figure Guide PDF' (https://www.embopress.org/page/journal/17574684/authorguide#figureformat).

3) At EMBO Press we ask authors to provide source data for the main figures. Our source data coordinator will contact you to discuss which figure panels we would need source data for and will also provide you with helpful tips on how to upload and organize the files.

4) A .docx formatted letter INCLUDING the reviewers' reports and your detailed point-by-point responses to their comments. As part of the EMBO Press transparent editorial process, the point-by-point response is part of the Review Process File (RPF), which will be published alongside your paper.

5) A complete author checklist, which you can download from our author guidelines (https://www.embopress.org/page/journal/17574684/authorguide#submissionofrevisions). Please insert information in the checklist that is also reflected in the manuscript. The completed author checklist will also be part of the RPF.

6) Please note that all corresponding authors are required to supply an ORCID ID for their name upon submission of a revised manuscript.

7) It is mandatory to include a 'Data Availability' section after the Materials and Methods. Before submitting your revision, primary datasets produced in this study need to be deposited in an appropriate public database, and the accession numbers and database listed under 'Data Availability'. Please remember to provide a reviewer password if the datasets are not yet public (see https://www.embopress.org/page/journal/17574684/authorguide#dataavailability).
In case you have no data that requires deposition in a public database, please state so in this section (This study includes no data deposited in external repositories). Note that the Data Availability Section is restricted to new primary data that are part of this study.

8) For data quantification: please specify the name of the statistical test used to generate error bars and P values, the number

(n) of independent experiments (specify technical or biological replicates) underlying each data point and the test used to calculate p-values in each figure legend. The figure legends should contain a basic description of n, P and the test applied. Graphs must include a description of the bars and the error bars (s.d., s.e.m.). Please provide exact p values.

9) Our journal encourages inclusion of *data citations in the reference list* to directly cite datasets that were re-used and obtained from public databases. Data citations in the article text are distinct from normal bibliographical citations and should directly link to the database records from which the data can be accessed. In the main text, data citations are formatted as follows: "Data ref: Smith et al, 2001" or "Data ref: NCBI Sequence Read Archive PRJNA342805, 2017". In the Reference list, data citations must be labeled with "[DATASET]". A data reference must provide the database name, accession number/identifiers and a resolvable link to the landing page from which the data can be accessed at the end of the reference. Further instructions are available at .

10) We replaced Supplementary Information with Expanded View (EV) Figures and Tables that are collapsible/expandable online. A maximum of 5 EV Figures can be typeset. EV Figures should be cited as 'Figure EV1, Figure EV2" etc... in the text and their respective legends should be included in the main text after the legends of regular figures.
- For the figures that you do NOT wish to display as Expanded View figures, they should be bundled together with their legends in a single PDF file called *Appendix*, which should start with a short Table of Content. Appendix figures should be referred to in the main text as: "Appendix Figure S1, Appendix Figure S2" etc.
- Additional Tables/Datasets should be labeled and referred to as Table EV1, Dataset EV1, etc. Legends have to be provided in a separate tab in case of .xls files. Alternatively, the legend can be supplied as a separate text file (README) and zipped together with the Table/Dataset file.
See detailed instructions here:

11) The paper explained: EMBO Molecular Medicine articles are accompanied by a summary of the articles to emphasize the major findings in the paper and their medical implications for the non-specialist reader. Please provide a draft summary of your article highlighting
- the medical issue you are addressing,
- the results obtained and
- their clinical impact.
This may be edited to ensure that readers understand the significance and context of the research. Please refer to any of our published articles for an example.

12) For more information: There is space at the end of each article to list relevant web links for further consultation by our readers. Could you identify some relevant ones and provide such information as well? Some examples are patient associations, relevant databases, OMIM/proteins/genes links, author's websites, etc...

13) Author contributions: CRediT has replaced the traditional author contributions section because it offers a systematic machine readable author contributions format that allows for more effective research assessment. Please remove the Authors Contributions from the manuscript and use the free text boxes beneath each contributing author's name in our system to add specific details on the author's contribution. More information is available in our guide to authors.

14) Disclosure statement and competing interests: We updated our journal's competing interests policy in January 2022 and request authors to consider both actual and perceived competing interests. Please review the policy https://www.embopress.org/competing-interests and update your competing interests if necessary.

15) Every published paper now includes a 'Synopsis' to further enhance discoverability. Synopses are displayed on the journal webpage and are freely accessible to all readers. They include a short stand first (maximum of 300 characters, including space) as well as 2-5 one-sentences bullet points that summarizes the paper. Please write the bullet points to summarize the key NEW findings. They should be designed to be complementary to the abstract - i.e. not repeat the same text. We encourage inclusion of key acronyms and quantitative information (maximum of 30 words / bullet point). Please use the passive voice. Please attach these in a separate file or send them by email, we will incorporate them accordingly.

Please also suggest a striking image or visual abstract to illustrate your article as a PNG file 550 px wide x 300-600 px high.

16) As part of the EMBO Publications transparent editorial process initiative (see our Editorial at http://embomolmed.embopress.org/content/2/9/329), EMBO Molecular Medicine will publish online a Review Process File (RPF) to accompany accepted manuscripts.
In the event of acceptance, this file will be published in conjunction with your paper and will include the anonymous referee reports, your point-by-point response and all pertinent correspondence relating to the manuscript. Let us know whether you agree with the publication of the RPF and as here, if you want to remove or not any figures from it prior to publication.
Please note that the Authors checklist will be published at the end of the RPF.

EMBO Molecular Medicine has a "scooping protection" policy, whereby similar findings that are published by others during

review or revision are not a criterion for rejection. Should you decide to submit a revised version, I do ask that you get in touch after three months if you have not completed it, to update us on the status.

I look forward to receiving your revised manuscript.

Yours sincerely,

Lise Roth

Lise Roth, PhD
Senior Editor
EMBO Molecular Medicine

***** Reviewer's comments *****

Referee #1 (Comments on Novelty/Model System for Author):

The manuscript contains a comprehensive data package and illustrated that adoptive cell therapy might benefit from addition of checkpoint blockade. Here demonstrated for NK cells. NKG2A as a checkpoint on NK cells has been described two decades ago and also Qa-1 as the ligand is old news. NK cell transfer as clinical approach for cancer treatment is under evaluation in clinical trials, but mostly via iv injection and not intratumoral as applied in this study. This limits clinical perspective of this study. In addition, few controls are lacking of the basic concept.

Referee #1 (Remarks for Author):

The paper by Melero et al reports a series of tumor treatment experiments in mouse models in which activated NK cells are injected intratumorally together with two blocking antibodies: to NKG2A and Qa-1. This combination led to delayed tumor outgrowth of MC38, CT26 and B16F10 tumors. Separately, these components were not capable of any tumor control. Allogeneic as well as syngeneic NK cells were comparably efficient and injected NK cells indeed lasted only a few days after transfer. Tumor control depended on CD8 T cells (ab-mediated depletion) and cDC1 (Batf3 ko mice), but not endogenous NK cells, indicating that the therapy provided a kickstart for endogenous adaptive immunity. The paper then shows combination therapy with aPD1, induction of protective memory and abscopal effect. Experiments in NSG mice showing effectiveness in human xenograft model with human NK cells and the clinical monalizumab Ab are completing the story.
The manuscript contains a comprehensive data package and illustrated that adoptive cell therapy might benefit from addition of checkpoint blockade. Here demonstrated for NK cells. NKG2A as a checkpoint on NK cells has been described two decades ago and also Qa-1 as the ligand is old news. NK cell transfer as clinical approach for cancer treatment is under evaluation in clinical trials, but mostly via iv injection and not intratumoral as applied in this study. This limits clinical perspective of this study. In addition, few controls are lacking of the basic concept.
Major issues:

1. isotypes of the treatment antibodies were not included in fig S2c in which blocking antibodies to NKG2A and Qa-1 were added in an in vitro lysis assay. The improvement in lysis is very small by addition of the antibodies. What ab concentration was used here? Include isotype control ab. More importantly, isotypes are missing in the in vivo tumor treatment experiments. Does NK injections with isotypes lead to delayed tumor control?
2. NK cells were injected intratumorally. This partly destroys the architecture and TME of the tumor. Were PBS injections with same volumes performed as control? It is possible that injections perse initiate antigen spread and cross-presentation to CD8 T cells which might be empowered by NKG2A checkpoint blockade.
3. Surprisingly, both aQa-1 and aNKG2A abs were required together with NK cell transfer to observe some tumor control. These two antibodies target the same axis, why is it needed to both sides of this axis?
4. How does human model work as NSG mice lack CD8 T cells and these were also not provided during the experiment? Should we conclude that the mouse models depend on CD8 T cells but the human model on direct effects by NK cells? Can this difference be explained?

Minor issues:
1. First paragraph of result section reads like M&M. rewrite
2. Qa-1b should be written with the b in superscript, since this indicates the allele variant.

3. Congeneric-> Congenic
4. Authors state that 'anti-Qa-1b mAb is a mouse IgG1 belonging to a subclass of Ig that does not elicit ADCC.' This is not correct. Mouse IgG1 isotype can bind to FcgRII and FcgRIII.
5. Fig 1c: How is delt with average tumor size in case of a drop-out due to sacrifice?

Referee #2 (Remarks for Author):

General summary
In this study, the authors demonstrate a synergistic role for combinatorial receptor/ligand blockade of HLA-E/NKG2A signaling for therapeutic intra-tumoral NK cell administration. Specifically, they show that anti-tumor activity was drastically improved when both HLA-E on tumors and NKG2A on intra-tumor injected NK cells were blocked, whereby this effect synergizes with systemic anti-PD1 therapy in syngeneic mouse cancer models, or with Cetuximab in a humanized mouse model of cancer.
This anti-tumor effect induced upon combined receptor/ligand blockade of HLA-E/NKG2A is mediated by conventional DCs and CD8 T cell responses in mice and is ADCC independent. The approach shows systemic and long-lasting effects when combined with anti-PD1 therapy.
In a xenografted human tumor model, direct NK cell-mediated ADCC could be enhanced by simultaneous blockade of NKG2A and EGFR (using cetuximab), leading to increased anti-tumor activity dependent on the degree of HLA-E expression on target tumor cells.
In general, the experiments are well-executed, the figures are clear, and the text reads well. The experiments performed include all necessary controls and the data provided are convincing and of good quality. Overall, the authors convincingly show the therapeutic efficacy of conjoint HLA-E/NKG2A blockade for treatment based on intra-tumoral delivery of effector NK cells, as well as the potential suitability of the approach in a clinal setting. Yet, I still have several comments on the current manuscript, which I listed below:

Major comments
• As indicated in the introduction, cancer therapy based on anti-NKG2A monoclonal antibody has already been tested in animal models and in the clinics and been showed to activate both NK and CD8+ T cells while combined with pharmaceutical inhibition of the PD-1 axis (or with EGFR inhibition using Cetuximab) (PMID: 30503213). Other studies have also shown the efficacy of NKG2A inhibition for cancer immunotherapy, for different types of solid tumors (PMID: 30503208; 35840162; 36099881). Although they indicate the specific setting in which the therapeutic approach they propose may be applied in the clinics, the authors should better highlight in the text the novel aspects of their study.
• Since monalizumab may induce adverse events in treated patients (PMID: 30503213), have the authors tried an in vitro blockade of NKG2A on activated NK cells before transfer of these cells into solid tumors? Is the effect comparable to intra-tumoral co-injection of NK cells with anti-NKG2A monoclonal antibody?
• Fig. 3 indicates that combinatorial receptor/ligand blockade of HLA-E/NKG2A signaling together with therapeutic intra-tumoral NK cell administration leads to increased accumulation of CD8+ T effectors and cDC1 antigen-presenting cells in tumor lesions. Is this simply a numbers game or are these immune cells also qualitatively affected by the treatment (e.g. via different expression of activation markers and/or production of effector molecules)?
• Along these lines, could the authors at least speculate on the type of cell death (death ligand / receptor interactions or via degranulation of granzyme / perforin-containing granules) induced by the double blockade and NK cell transfer treatment in their models? This question is relevant as the type of cell death (induced by activated NK cells) influences tumor immunogenicity (PMID: 36864446; 29275465, e.g.).

Minor comments
• NK-cell mediated immunogenic cell death have been described by studies before the ones reported in the introduction: PMID: 19406986; 14563320.
• Fig 4C; the experimental group with anti-NK1.1 treatment only is not displayed in the graph.
• Fig 7F: administration of NK cells apparently leads to an increase size of HLA-E high tumor in this experiment. This is in contrast with the clear-cut data of the other models / experiments. Is this simply due to biologic variation? Or rather related to the specific model that was used? In addition, the authors could assess by a statistical analysis if there is indeed -- as they claim in the text -- increased anti-tumor response in HLA-E high versus low tumors for the setting presented in this figure panel. This could be done e.g. by comparing the percent of NK cell-mediated killing for HLA-E high versus low tumors.
• Fig. 7; panel C shows both single and combined treatments with monalizumab and/or cetuximab; panels D-F depicts treatment with monalizumab only (versus NK cell only or control). Therefore, the author may consider swapping the order of these panels.
• Page 20: the formula indicated to calculate tumor volume seems to be incorrect (typo?).
• Fig 1D: do these data present tumor-free or surviving / non-euthanized mice? Along these lines, and in relation to Fig. 5D: do the animals in their model either entirely clear the tumor cells or show tumors that keep growing (i.e. not state of equilibrium with persisting tumors of unchanging size)? This information could be indicated in the methods.

<u>POINT BY POINT REPLY</u>

<u>Referee #1</u>

The paper by Melero et al reports a series of tumor treatment experiments in mouse models in which activated NK cells are injected intratumorally together with two blocking antibodies: to NKG2A and Qa-1. This combination led to delayed tumor outgrowth of MC38, CT26 and B16F10 tumors. Separately, these components were not capable of any tumor control. Allogeneic as well as syngeneic NK cells were comparably efficient and injected NK cells indeed lasted only a few days after transfer. Tumor control depended on CD8 T cells (ab-mediated depletion) and cDC1 (Batf3 ko mice), but not endogenous NK cells, indicating that the therapy provided a kickstart for endogenous adaptive immunity. The paper then shows combination therapy with aPD1, induction of protective memory and abscopal effect. Experiments in NSG mice showing effectiveness in human xenograft model with human NK cells and the clinical monalizumab Ab are completing the story.
The manuscript contains a comprehensive data package and illustrated that adoptive cell therapy might benefit from addition of checkpoint blockade. Here demonstrated for NK cells. NKG2A as a checkpoint on NK cells has been described two decades ago and also Qa-1 as the ligand is old news. NK cell transfer as clinical approach for cancer treatment is under evaluation in clinical trials, but mostly via iv injection and not intratumoral as applied in this study. This limits clinical perspective of this study. In addition, few controls are lacking of the basic concept.

Response 1: Thank you for accurately summarizing our work and the overall positive appraisal of it. Regarding applicability for translation, the availability of GMP allogeneic NK cells and monalizumab (collaboration with AstraZeneca) make clinical

translation realistic and the point of injecting part of the NK cells intratumorally is on the table in that regard.

Major issues:

1. *Isotypes of the treatment antibodies were not included in fig S2c in which blocking antibodies to NKG2A and Qa-1 were added in an in vitro lysis assay. The improvement in lysis is very small by addition of the antibodies. What ab concentration was used here? Include isotype control ab. More importantly, isotypes are missing in the in vivo tumor treatment experiments. Does NK injections with isotypes lead to delayed tumor control?*

Response 2: In in-vitro and in-vivo experiments, isotype-matched controls were actually used for anti-NKG2A and anti-$Qa1^b$, as it was already mentioned in the Material and Methods' section in our original submission. However, in light of the reviewer's comment, this needs to be better clarified in the text and figures. That is why we have added the information regarding the isotype control treatments for each condition in all the corresponding figures and figure legends pertaining the alluded in-vivo and in-vitro experiments.

For the cytotoxicity assays displayed in appendix figure 2S, as indicated in the Material and Methods' section of the original submission, the NK cells and tumor cells were incubated with 10 µg/mL of anti-NKG2A and 5 µg/mL of anti-$Qa1^b$ respectively for 30 min at 4ºC and washed prior to the co-cultures in the cytotoxicity assays. Isotype controls were used where needed and it is now more clearly indicated in the new Appendix Figure 2S.

As suggested, we have repeated this experiment using a chromium-release cytotoxicity assay, which tends to be more sensitive than CFSE-based killing assays. We have obtained similar results that conclude that the dual blockade of NKG2A and $Qa1^b$ is better than the use of individual mAbs in these conditions as shown in the Figure 1 shown at the end of this document for reviewer's inspection.

In the case of experiments in tumor-bearing mice, all groups receiving intratumoral injections of NK cells were also treated with the corresponding isotype controls for anti-NKG2A and anti-$Qa1^b$ to rule out possible unspecific artefacts. We have made our best to make this point clear for the readers, especially in the figure legends. Additional mention to the control isotypes used can be found throughout the main text (page 8 line 206, page 10 line 253, page 11 line 286, page 14 line 353)

2. *NK cells were injected intratumorally. This partly destroys the architecture and TME of the tumor. Were PBS injections with same volumes performed as control? It is possible that injections perse initiate antigen spread and cross-presentation to CD8 T cells which might be empowered by NKG2A checkpoint blockade.*

Response 3: As detailed in Response 2, control mice received 50 µL of PBS supplemented with mIgG1 (day 5) and rIgG (day 6) and therefore it is expected that the tissue architecture destruction upon injection of the tumors in control mice would be similar to the one experienced in the other groups, which were also injected with anti-$Qa1^b$/mIgG; NK and/or anti-NKG2A/rIgG on days 5 and 6.

Nevertheless, to evaluate the degree of histological damage due to intratumoral injections, we performed an experiment where we injected once with 50 µL of PBS into MC38 tumors on days 5 or 8 post-tumor inoculation and tumors were collected four and seven days post-tumor injections, respectively. H&E stained tumor sections did not

show any meaningful pathological damage of PBS-injected tumors when compared with non-injected tumors at the two time-points analyzed (3 or 7 days post-IT injections). These results are shown in the Figure 2 at the end of this document for the reviewer's evaluation.

3. *Surprisingly, both aQa-1 and aNKG2A abs were required together with NK cell transfer to observe some tumor control. These two antibodies target the same axis, why is it needed to both sides of this axis?*

Response 4: We were also surprised by these findings. However, we only have the empirical evidence. We have consistently observed in both in vitro and in vivo experiments that the dual blockade of $Qa1^b$ and NKG2A seems to be somehow better than the individual blockade of each component of the NKG2A inhibitory pathway. One possible explanation could be that the dosage administered is not sufficient to block each individual component and thus higher doses of one of them could be sufficient to abrogate the NKG2A inhibitory pathway.

To test this hypothesis, we first performed an in vitro cytotoxicity assay in which tumors and activated NK cells separately pre-incubated with anti-$Qa1^b$/mIgG or anti-NKG2A/rIgG were co-cultured in the presence of mAb at different concentrations (shown in Figure 1 at the end of this document for reviewer's evaluation). We found that the addition of more anti-$Qa1^b$ and/or anti-NKG2A (or their corresponding isotype controls) did not meaningfully increased the percentage of lysis of IFNγ-treated tumors if the tumor cells or NK cells were pre-incubated with anti-$Qa1^b$ or anti-NKG2A, respectively, as the concentration of the mAb used for the pre-incubations seemed to be above the saturation level of the target (green columns). However, a slight, but not significant, increase of tumor lysis was observed by the addition of anti-NKG2A to the

cultures of rIgG-treated NK cells and anti-$Qa1^b$ treated tumors (red columns), whereas the addition of anti-$Qa1^b$ to the cultures of anti-NKG2A-treated and mIgG1-treated tumors did not have such effect (violet columns). Similarly, the addition of anti-$Qa1^b$ to the cultures of rIgG-treated NK cells and mIgG1-treated tumors (blue columns) had less effect than the addition of anti-NKG2A. This data suggest that the blockade of NKG2A is more effective than the blockade of anti-$Qa1^b$ in this setting. However, as implied by the reviewer, these results are not sufficient to confirm the reason for the greater combined effect due to the variability of the results, the use of saturation concentrations of the mAbs and the small increase of tumor lysis observed when dual blockade was performed in ex-vivo NK-cell cytotoxicity experiments.

This is why we have performed an in vivo experiment in which we compared the MC38 tumor growth of mice treated with high dose of anti-NKG2A (100 µg) and mice treated with low dose of anti-NKG2A (30µg) and anti-$Qa1^b$ (30µg) (New Appendix Figure S6). We found that the intratumoral injection of 100 µg anti-NKG2A led to a delay of tumor growth when compared to control-treated mice. However, the anti-tumor response was still more pronounced in the group that received intratumoral NK cells along with 30 µg doses of anti-NKG2A and anti-$Qa1^b$. Because of the results obtained in vivo in the in-vitro assay, we did not test groups receiving high doses of anti-$Qa1^b$. Overall, these results confirm the synergy between NK cells and lower doses of anti-NKG2A/$Qa1^b$, and exclude the hypothesis that higher doses of anti-NKG2A might overcome the of the need for the dual NKG2A and $Qa1^b$ blockade when given in the presence of exogenous activated NK cells. This is discussed with due caution in the revised version of the manuscript since this would have implications for double NKG2A and HLA-E blockade in humans (Appendix Figure S6, page 9 line 230, page 16 line 401).

4. *How does human model work as NSG mice lack CD8 T cells and these were also not provided during the experiment? Should we conclude that the mouse models depend on CD8 T cells but the human model on direct effects by NK cells? Can this difference be explained?*

Response 5: The xenografted mouse model used Rag2$^{-/-}$IL2R$\gamma^{-/-}$ imunodeficient mice, as the reviewer's has pointed out. Therefore, we cannot evaluate the impact of CD8 T cells on the anti-tumor effects mediated by intratumoral co-injections of human NK cells and monalizumab due to the fact that there are not human CD8 T cells in the system. This model, however, measures the ability of human NK cells (with or without monalizumab) to directly eliminate tumor cells. Of note, at difference with the syngeneic mouse tumor models, no complete tumor rejection was observed in any condition in the xenografted mice, perhaps indicating that direct tumor lysis mediated by NK cells is insufficient by its own to completely eliminate tumors. In order to evaluate the contribution of CD8 T cells, we would have to intravenously inject human CD8 T cells or peripheral blood lymphocytes prior to intratumoral injections of NK cells and/or monalizumab. Unfortunately, the appearance of GvHD mediated by human T cells shortly after T-cell infusion prevents the use of this model to fully determine the role of human CD8 T cells in the therapeutic efficacy of NK + monalizumab treatments. Moreover, the cDC cross-presenting subset of DC would not be reconstituted in such models. Hence, we discuss this limitation of the humanized model clearly indicating that it does not explore the ensuing adaptive immunity component for the therapeutic effects (page 13 line 342)

Minor issues:

1. *First paragraph of result section reads like M&M. rewrite*

Response 6: This paragraph has been rewritten as per the reviewer's suggestion (page 8 line 188).

2. *Qa-1b should be written with the b in superscript, since this indicates the allele variant.*

Response 7: Qa-1b has been changed to appear like Qa-1^b in the manuscript per reviewer's suggestion.

3. *Congeneric-> Congenic*

Response 8: We thank the reviewer for picking up this error. "Congeneric" has been changed for "congenic" (page 11 line 271).

4. *Authors state that 'anti-Qa-1b mAb is a mouse IgG1 belonging to a subclass of Ig that does not elicit ADCC.' This is not correct. Mouse IgG1 isotype can bind to FcgRII and FcgRIII.*

Reponses 9: We thank the reviewer for his/her comment. We have changed this sentence and it now includes the fact that mIgG1 can bind to FcgRII and FcgRIII (page 9 line 224).

5. *Fig 1c: How is delt with average tumor size in case of a drop-out due to sacrifice?*

Response 10: When tumor size reached 15mm of diameter or mice presented signs of distress per ECAE guidelines, the animals were sacrificed. The tumor size of the sacrificed mice were not dragged in the average tumor size once they were sacrificed. This is why the average tumor size graph display is not shown beyond 28 days post-tumor inoculation for MC38 tumor mouse models since this is the last day when there are control mice alive. The same it is true for the other tumor mouse models, although the last shown tumor sizes vary in function of the speed of tumor progression in each model (21 days for B16.OVA and 24 days for CT26). This information has been included in the Material and Methods' section (page 23 line 567)

Referee #2

General summary

In this study, the authors demonstrate a synergistic role for combinatorial receptor/ligand blockade of HLA-E/NKG2A signaling for therapeutic intra-tumoral NK cell administration. Specifically, they show that anti-tumor activity was drastically improved when both HLA-E on tumors and NKG2A on intra-tumor injected NK cells were blocked, whereby this effect synergizes with systemic anti-PD1 therapy in syngeneic mouse cancer models, or with Cetuximab in a humanized mouse model of cancer.

This anti-tumor effect induced upon combined receptor/ligand blockade of HLA-E/NKG2A is mediated by conventional DCs and CD8 T cell responses in mice and is ADCC independent. The approach shows systemic and long-lasting effects when combined with anti-PD1 therapy.

In a xenografted human tumor model, direct NK cell-mediated ADCC could be

enhanced by simultaneous blockade of NKG2A and EGFR (using cetuximab), leading to increased anti-tumor activity dependent on the degree of HLA-E expression on target tumor cells.

In general, the experiments are well-executed, the figures are clear, and the text reads well. The experiments performed include all necessary controls and the data provided are convincing and of good quality. Overall, the authors convincingly show the therapeutic efficacy of conjoint HLA-E/NKG2A blockade for treatment based on intra-tumoral delivery of effector NK cells, as well as the potential suitability of the approach in a clinical setting. Yet, I still have several comments on the current manuscript, which I listed below:

Response 11: Our work is well summarized by the reviewer and we are grateful for the overall positive comments on our experimentation.

Major comments:

1. *As indicated in the introduction, cancer therapy based on anti-NKG2A monoclonal antibody has already been tested in animal models and in the clinics and been showed to activate both NK and CD8+ T cells while combined with pharmaceutical inhibition of the PD-1 axis (or with EGFR inhibition using Cetuximab) (PMID: 30503213). Other studies have also shown the efficacy of NKG2A inhibition for cancer immunotherapy, for different types of solid tumors (PMID: 30503208; 35840162; 36099881). Although they indicate the specific setting in which the therapeutic approach they propose may be applied in the clinics, the authors should better highlight in the text the novel aspects of their study.*

Reponses 12: We thank the reviewer for his/her comment. In order to emphasize the importance of the local delivery of NK cells to the tumor site for our experimental settings, we performed an experiment where NK cells were given either intratumorally or intravenously and tumor growth and survival were evaluated (Appendix Figure S4 of the revised version). As expected, an anti-tumor effect of the combined treatment of NK cells and anti-NKG2A/$Qa1^b$ was only obtained when the NK cells were administered intratumorally, highlighting the importance of the intratumoral delivery to exploit in situ NK-cell cytotoxic potential. Accordingly, in the revised discussion we highlighted the novelty of the approach (page 17 line 438) .

New Appendix Figure S4 has been added (page 9 line 219).

2. *Since monalizumab may induce adverse events in treated patients (PMID: 30503213), have the authors tried an in vitro blockade of NKG2A on activated NK cells before transfer of these cells into solid tumors? Is the effect comparable to intra-tumoral co-injection of NK cells with anti-NKG2A monoclonal antibody?*

Response 12: This is an excellent suggestion. According to the results presented by Andre and collaborators of the phase II clinical trial on SCCHN patients mentioned by the reviewer (NCT 02643550 PMID: 30503213), the iv administration of monalizumab, alone or combined with cetuximab, caused adverse events (AEs) in 93% of the cases, but those AEs were of grades 1-2 , rapidly reversible and easily manageable. Because in the proposed immunotherapeutic approach, anti-NKG2A is given at low dose intratumorally, it is not expected that intratumoral anti-NKG2A treatment would cause significant AEs. As suggested by the reviewer, we evaluated pre-incubation of anti-NKG2A before NK-cell infusion to see if it causes comparable anti-tumor responses similar to intratumoral administration of free mAb. This experiment demonstrated that

this is actually the case as shown in the Appendix Figure S9. Interestingly, the intratumoral administration of anti-$Qa1^b$ along with anti-NKG2A pre-incubated NK cells had stronger and more durable control of tumor growth when compared to mice treated only with anti-NKG2A pre-incubated NK cells, highlighting once more the synergy that seems to exist between anti-NKG2A and anti-$Qa1^b$ mAbs. This new data has been added as Appendix Figure S9 (Page 11 line 284).

3. *Fig. 3 indicates that combinatorial receptor/ligand blockade of HLA-E/NKG2A signaling together with therapeutic intra-tumoral NK cell administration leads to increased accumulation of CD8+ T effectors and cDC1 antigen-presenting cells in tumor lesions. Is this simply a numbers game or are these immune cells also qualitatively affected by the treatment (e.g. via different expression of activation markers and/or production of effector molecules)?*

Response 13: In order to answer this reviewer's question, we evaluated in a functional assay if effector CD8 T cells were qualitatively enhanced by the proposed therapeutic treatment. Tumor infiltrated lymphocytes were collected from MC38-tumor bearing mice seven days after completing IT treatment (Appendix Figure S7A) and tumor-derived cell suspensions were stimulated with PMA/Ionomycin for 4h in the presence of monensin. The results displayed on Appendix Figure S7 showed that CD8 T cells from IT-treated mice had a significant increase in the degranulation marker CD107a on their surface when compared to CD8 T cells obtained from control-treated mice (Appendix Figure S7B-C). However, no significant differences were observed between the NK and/or anti-NKG2A/$Qa1^b$ treated mice. With regard the ability to secrete IFNγ, although a slight increase in the percentage of IFNγ+ CD8 T cells could be detected on NK + anti-NKG2A/$Qa1^b$ treated mice (Appendix Figure S7D-E), only a non-statistically

significance trend was observed when comparing the groups. In addition, the proportion of $CD44^+CD62L^-$ effector CD8 T cells or their ability to proliferate, measured by the proliferation marker Ki67, was similar in all the groups (data not shown). These results indicate that the anti-tumor efficacy obtained by the NK + anti-NKG2A/$Qa1^b$ IT treatment is mostly due to an increase in the number of CD8 T cells that do not seem to be significantly more functional. Appendix Figure S7 has been added (page 10 line 255). The scanty numbers of intratumoral cDC1 cells precluded comparative functional assessments

4. *Along these lines, could the authors at least speculate on the type of cell death (death ligand / receptor interactions or via degranulation of granzyme / perforin-containing granules) induced by the double blockade and NK cell transfer treatment in their models? This question is relevant as the type of cell death (induced by activated NK cells) influences tumor immunogenicity (PMID: 36864446; 29275465, e.g.).*
Response 14: This point is very well taken. In previous studies from our group (Minute et al., JITC 2020), we had shown that NK-cell cytotoxicity showed features of immunogenic cell death (calreticulin exposure, ATP and HMGB1 release) and resulted in tumoral immunogenicity in mice. Hence, we discuss the point with the suggested references. Moreover, analysis of the tumor microenvironment in our experimental settings following intratumoral treatment. In line with Minute and collaborators' work, higher levels of calreticulin exposure and activated caspase 3 were detectable in the retrieved MC-38 tumor cells treated with intratumoral NK + anti-NKG2A/$Qa1^b$. These results are included in the new Appendix Figure S10 (page 12 line 300) and discussed in the discussion section (page 16 line 411).

Minor comments

1. *NK-cell mediated immunogenic cell death have been described by studies before the ones reported in the introduction: PMID: 19406986; 14563320.*

Response 15: We thank the reviewer for his/her comments. These references have been included in the manuscript (page 16 line 414 and line 419).

2. *Fig 4C; the experimental group with anti-NK1.1 treatment only is not displayed in the graph.*

Response 16: We truly thank the reviewer for detecting this error. When introducing the data for the original figure, the data of rIgG (violet square) was incorrectly added to the data of anti-CD4 (blue diamond), which then resulted in incorrectly displaying data for 12 mice of two different groups, and the data of anti-NK1.1 (orange square) was displayed by mistake in the rIgG control group (violet square). This has now been corrected and all the groups are correctly labeled in the new figure (Figure 4). We apologize for this mistake.

3. *Fig 7F: administration of NK cells apparently leads to an increase size of HLA-E high tumor in this experiment. This is in contrast with the clear-cut data of the other models / experiments. Is this simply due to biologic variation? Or rather related to the specific model that was used?*

Response 17: As the reviewer points out, we believe that biological variability accounts for the differences seen in Fig. 7F and Fig. 7D (new Fig. 7C and Fig. 7E). In the tumor mouse models, we used inbred mice, which significantly reduced the variability

between experiments, whereas in the humanized mouse xenograft models, we rely on healthy volunteers as a source for NK cells, which are highly variable and might respond differently to the expansion and activation protocols. Differences in the tumor growth of engrafted Cal-27 cells on immunodeficient mice between experiments could also account for the variability observed between both experiments.

4. *In addition, the authors could assess by a statistical analysis if there is indeed -- as they claim in the text -- increased anti-tumor response in HLA-E high versus low tumors for the setting presented in this figure panel. This could be done e.g. by comparing the percent of NK cell-mediated killing for HLA-E high versus low tumors.*

Response 18: per reviewer's suggestion, a statistical analysis was performed to confirm the increased in vivo anti-tumor response obtained by NK + monalizumab treatment in HLA-E^{high} tumors. To highlight this difference, in figure 3C for reviewer shown at the end of the document, we compared the tumor volume of NK + monalizumab treatment for HLA-E^{low} against the one observed for HLA-E^{high} tumors. In the revised Figure 7D, this statistical difference is highlighted in red.

We also included data showing the percentage of NK cell-mediated in vitro killing against HLA-E^{low} and HLA-E^{high} tumors (New Figure 7F). Although no statistical differences are found in the total percentage of tumor lysis of monalizumab-treated NK cells between HLA-E^{low} and HLA-E^{high} tumors, it is important to emphasize that the baseline NK cell-mediated lysis is higher for HLA-E^{low} tumors than HLA-E^{high} tumors (statistical differences are now highlighted in red) and consequently, the contribution of monalizumab +/- cetuximab to the NK cell-mediated tumor lysis is more pronounced in tumors with higher expression levels of HLA-E. This is shown for reviewer's inspection

in Figure 3D shown at the end of this document and has been included in the manuscript in Figure 7 (page 14 line 360).

5. *Fig. 7; panel C shows both single and combined treatments with monalizumab and/or cetuximab; panels D-F depicts treatment with monalizumab only (versus NK cell only or control). Therefore, the author may consider swapping the order of these panels.*

Response 19. We have followed the reviewer's suggestion and changed the order of panels in the figure (revised figure 7).

6. *Page 20: the formula indicated to calculate tumor volume seems to be incorrect (typo?).*

Response 20: The formula has been corrected

7. *Fig 1D: do these data present tumor-free or surviving / non-euthanized mice? Along these lines, and in relation to Fig. 5D: do the animals in their model either entirely clear the tumor cells or show tumors that keep growing (i.e. not state of equilibrium with persisting tumors of unchanging size)? This information could be indicated in the methods.*

Response: In all the figures depicting percentage of survival, the final surviving mice represent the mice which survived due to complete rejection of the tumors with experiments ending at the indicating time-points without any sings of tumor regrowth. This is clarified in MM's section (page 9 line 216, page 13 line 319)

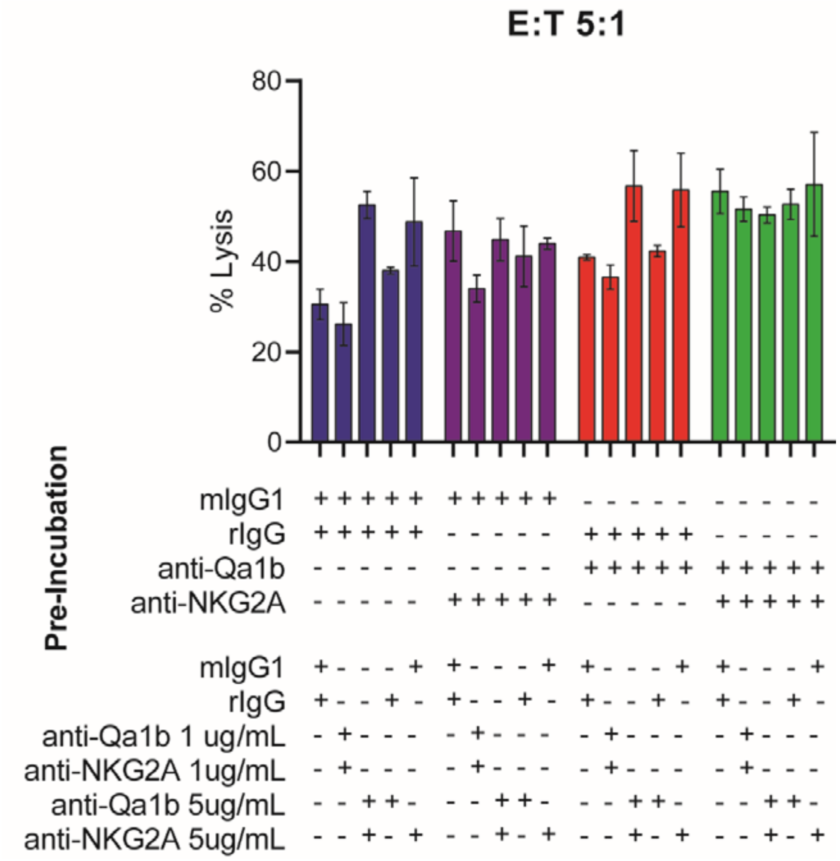

Figure 1 for Reviewers. The dual blockade of Qa1^{b} and NKG2A increases NK cell-mediated tumor lysis in a Chromium release cytotoxic assay. IFNγ-treated MC38 tumor cells and IL-15 activated NK cells were pre-incubated with 5 μg/mL or 10 μg/mL of anti-Qa1^{b}, anti-NKG2A or corresponding isotype controls for 30 min at 4ºC and then

washed twice with PBS. Cr^{51}-loaded tumor cells were then co-culture with NK cells along with different doses of the indicated free mAb for 4 hours. The total percentage of tumor lysis at an effector:target ratio of 5:1 is shown for each condition. Data represents an individual experiment done in triplicate and two-way ANOVA were used to assess significance.

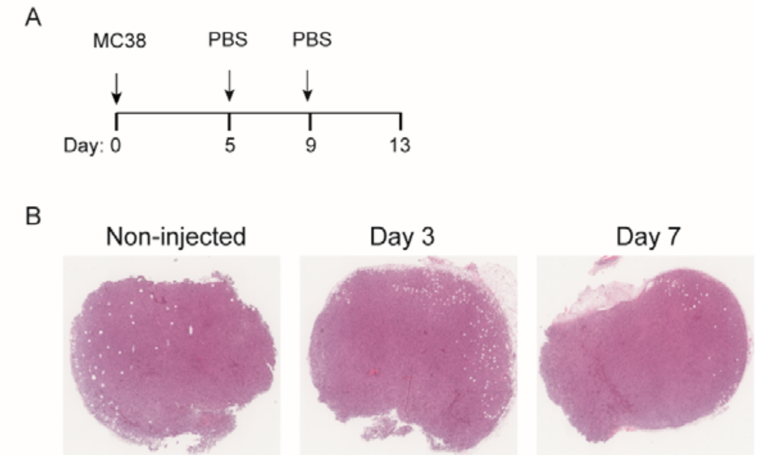

Figure 2 for Reviewers. No meaningful histological damage was observable in the TME after intratumoral injection of PBS as compared to non-injected tumors. (A) Schematic representation of the regimen followed for intratumoral injections. Briefly, C57BL/6 mice were subcutaneously injected with $4x10^5$ MC38 cells in the right flank. When tumors reach approximately 80 mm^3 at day 6, some mice were injected intratumorally with 50 µL of PBS. On day 8, other mice received 50 uL intratumoral injections of PBS. All tumors were collected at day 13 (4 and 7 days post-intratumoral injections) and fixed samples in 10% neutral-buffered formalin (24h) and ethanol 70% (48h) were embedded in paraffin and stained for H&E. **(B)** Representative images of histopathological 3 um-thick sections stained with H&E for non-injected tumors and

injected tumors at day 4 and 7 post-injections at 20x magnification using Leica Aperio CS2 microscope.

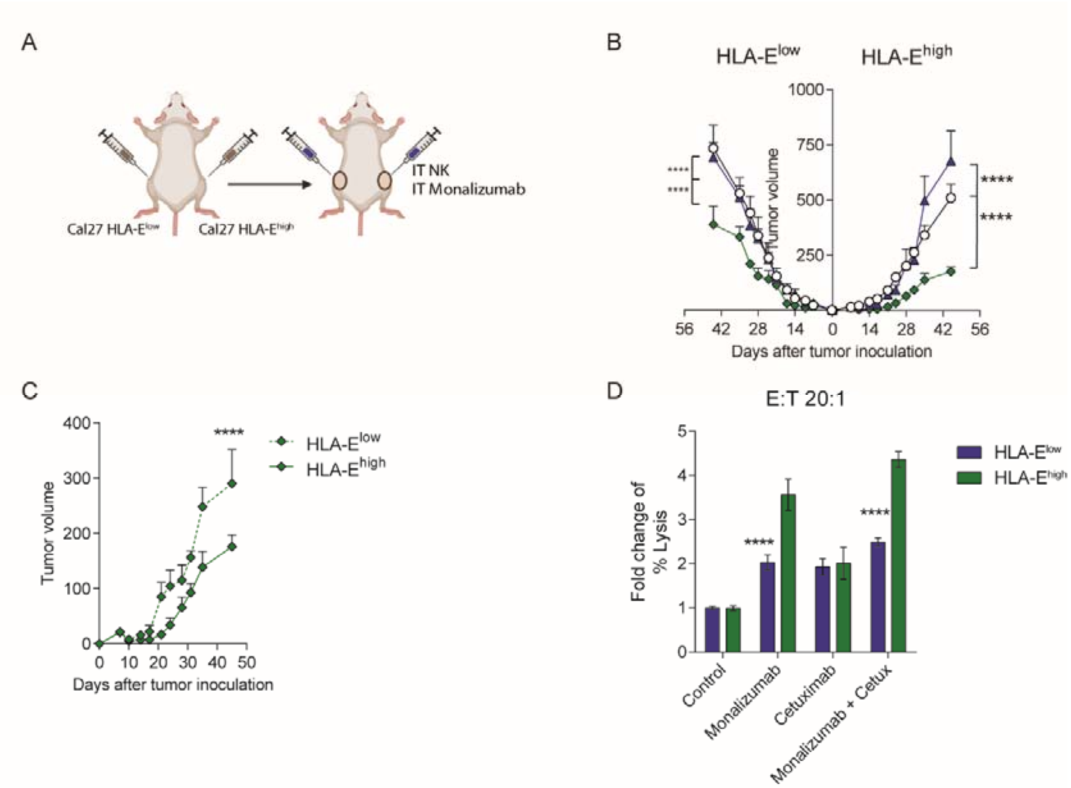

Figure 3 for Reviewers. The synergistic effect of NK cells and monalizumab is more prominent in HLA-E^{high} tumors. Data from Figure 7 from intratmoral NK and monalizumab treatment is represented in a different way to highlight the differences in the control of tumor growth between tumors expressing different levels of HLA-E. **(A)** Schematic representation of the dose regimen followed. **(B)** The progression tumor volume (mm^3) is shown over time for each treatment condition for HLA-E^{low} (left) and HLA-E^{high} (right) tumors. **(C)** The average tumor volume for tumors receiving NK and monalizumab intratumoral injections is shown for HLA-E^{low} and HLA-E^{high} tumors. **(D)**

Fold change increase of tumor lysis for HLA-E^{low} and HLA-E^{high} tumors after normalizing the data to the lysis obtained of NK cells against HLA-E^{low} and HLA-E^{high} tumors respectively (control). Data were fitted to a third-order polynomial and compared using Extra sum-of-squares F test. In B, significant differences are displayed for comparisons of each group with the NK + monalizumab group (***p<0.001).

12th Aug 2023

Dear Dr. Melero,

Thank you for the submission of your revised manuscript to EMBO Molecular Medicine. We have now received the reports from the 2 referees who re-reviewed your manuscript. As you will see, they are supportive of publication pending minor revisions, and we will therefore be able to accept your manuscript once the following points will be addressed:

1/ Referees' comments: please address the remaining issue raised by referee #2.

2/ Main manuscript text:
- Please address the queries from our data editors in the related Data Edited manuscript file. Remove the yellow highlights in the text and only keep in track changes mode any new modification.
- We can accommodate a maximum of 5 keywords, please adjust accordingly.
- Materials and methods:
o Mice: please indicate the housing/husbandry conditions (night/light cycle, access to food and water).
o Human material: please include a statement that informed consent was obtained from all subjects and that the experiments conformed to the principles set out in the WMA Declaration of Helsinki and the Department of Health and Human Services Belmont Report.
o Antibodies: please provide dilutions/concentrations.
o Statistics: please include information on blinding, randomization, inclusion/exclusion criteria.
- Data Availability: Please move the data availability section at the end of the Materials and Methods, and only include primary datasets produced in this study and deposited in an appropriate public database. In case you have no data that requires deposition in a public database, please state so in this section: "This study includes no data deposited in external repositories."
- Acknowledgements: please merge the funding with the acknowledgements. All funding sources should also be added to the submission system, and match the information provided in the manuscript.
- Author contributions: CRediT has replaced the traditional author contributions section because it offers a systematic machine-readable author contributions format that allows for more effective research assessment. Please remove the Authors Contributions from the manuscript and use the free text boxes beneath each contributing author's name in our system to add specific details on the author's contribution. More information is available in our guide to authors.
- Please rename to the conflict of interest to "Disclosure statement and competing interests". We updated our journal's competing interests policy and request authors to consider both actual and perceived competing interests. Please review the policy https://www.embopress.org/competing-interests and update your competing interests if necessary.

3/ Figures and Appendix:
- Please provide exact p values (not a range) in the figures or their legends, including for ns (non-significant) p values.
- Please note that you have the possibility to make some of your Appendix figures Expanded View (EV) Figures and Tables that are collapsible/expandable online. A maximum of 5 EV Figures can be typeset. EV Figures should be cited as 'Figure EV1, Figure EV2" etc... in the text and their respective legends should be included in the main text after the legends of regular figures. The remaining figures should be bundled together with their legends in a single PDF file called *Appendix*, which should start with a short Table of Content. In the Appendix file, please remove the yellow highlights and place each figure legends under the corresponding figure.
- Make sure all your figures are referenced in the manuscript text. A callout is currently missing for Fig. 2E.

4/ At EMBO Press we ask authors to provide source data for the main figures. Our source data coordinator will contact you to discuss which figure panels we would need source data for and will also provide you with helpful tips on how to upload and organize the files.

5/ Checklist: please indicate in the right column where the information can be found in the manuscript. Please provide information in the "Experimental study design and Statistics" section, and in general adjust the checklist to match the information provided in the Materials and Methods.

6/ Synopsis: Thank you for providing a synopsis text and picture.
- Please upload the synopsis text as a separate document. It should include a short stand-first (maximum 300 characters) and 2-5 one-sentence bullet points that summarizes the study.
- Please resize the picture as a png file 550 pixels wide x 200-400 pixels high, and make sure that the text remains legible.

7/ As part of the EMBO Publications transparent editorial process initiative (see our Editorial at http://embomolmed.embopress.org/content/2/9/329), EMBO Molecular Medicine will publish online a Review Process File (RPF) to accompany accepted manuscripts.
This file will be published in conjunction with your paper and will include the anonymous referee reports, your point-by-point

response and all pertinent correspondence relating to the manuscript. Let us know whether you agree with the publication of the RPF and as here, if you want to remove or not any figures from it prior to publication.
Please note that the Authors checklist will be published at the end of the RPF.

I look forward to receiving your revised manuscript.

Yours sincerely,

Lise Roth

Lise Roth, PhD

Senior Editor

EMBO Molecular Medicine

***** Reviewer's comments *****

Referee #1 (Comments on Novelty/Model System for Author):

medium: lack of novel mechanism or therapeutic target.

Referee #1 (Remarks for Author):

satisfied with the provided additional data and clarification.

Referee #2 (Remarks for Author):

The authors have added several new convincing data that strengthen the manuscript. Almost all my comments were adequately addressed. Yet, there is still an unresolved issue, which is detailed below.

My feedback on responses 12a:

>The novel experiment comparing the different route of injection convincingly demonstrate a more efficient anti-tumor response upon intra-tumoral delivery of the NK cells.
>In the revised discussion, the authors indeed indicate the translational aspect of their work. Yet the text highlighted in the manuscript (lines 444-451 / page 18 in the .pdf document and lines 438-445 / page 17 in the .docx document) is unchanged compared to the previous manuscript version.

The authors addressed the editorial issues.

1st Sep 2023

Dear Dr. Melero,

Thank you for submitting your revised files, and please accept my apologies for the delay in getting back to you as I was away from the office.
I appreciate that you have addressed most of our editorial queries, however some pending issues remain. Therefore, before I can proceed with acceptance of the manuscript, please address the following:

1. Please address the queries from our data editors in the related Data Edited manuscript file. In case you did not find this file, I paste below the comment from the data editor, relating to Figure 7:
"Please define the number and the nature of the replicates in C and E. Please define the statistical test in C."
2. Data Availability: please remove "All primary data will be made available upon reasonable request to the corresponding authors."
3. At EMBO Press we ask authors to provide source data for the main figures. Our source data coordinator has contacted you on August 15th with a checklist. Please find the checklist also attached to this email for your convenience. We would kindly ask you to submit the requested source data with the completed checklist.
4. Thank you for providing a synopsis. I introduced minor modifications to fit our style and format, let me know if you agree with the following or amend as you see fit:
An approach that involves intratumoral injections of NK cells together with neutralizing antibodies against the NKG2A inhibitory receptor was developed, aiming at extending the efficacy of NK cell-based immunotherapies to solid malignances.
• Intratumoral co-injection of pre-activated NK cells and NKG2A neutralizing antibodies achieves synergistic anti-tumor activity against solid mouse tumor models
• CD8 T cell- and cDC1 responses mediate the anti-tumor efficacy of intratumoral NK and anti-NKG2A/Qa-1b mAb administrations
• Systemic anti-PD-1 mAb injections synergistically enhance the efficacy of intratumoral NK cells + anti-NKG2A/Qa-1b mAbs
• Intratumoral delivery of human NK cells and monalizumab (anti-NKG2A) to xenografted mouse tumors exerts synergistic effects

Please accept the previous changes before resubmission, and only leave in track changes mode any new modification.

Thank you for bearing with these last changes. I look forward to receiving your revised manuscript as soon as possible.

Yours sincerely,

Lise Roth

Lise Roth, PhD
Senior Editor
EMBO Molecular Medicine

The authors addressed the remaining editorial issues.

7th Sep 2023

Dear Dr. Melero,

I am pleased to inform you that your manuscript is accepted for publication and is now being sent to our publisher to be included in the next available issue of EMBO Molecular Medicine!

We would like to remind you that as part of the EMBO Publications transparent editorial process initiative, EMBO Molecular Medicine will publish a Review Process File online to accompany accepted manuscripts. If you do NOT want the file to be published or would like to exclude figures, please immediately inform the editorial office via e-mail.

Please read below for additional IMPORTANT information regarding your article, its publication and the production process.

Congratulations on your interesting work,

With kind regards,

Lise Roth

Lise Roth, Ph.D
Senior Editor
EMBO Molecular Medicine

Follow us on Twitter @EmboMolMed
Sign up for eTOCs at embopress.org/alertsfeeds

*** *** *** IMPORTANT INFORMATION *** *** ***

SPEED OF PUBLICATION
The journal aims for rapid publication of papers, using using the advance online publication "Early View" to expedite the process: A properly copy-edited and formatted version will be published as "Early View" after the proofs have been corrected. Please help the Editors and publisher avoid delays by providing e-mail address(es), telephone and fax numbers at which author(s) can be contacted.

Should you be planning a Press Release on your article, please get in contact with embomolmed@wiley.com as early as possible, in order to coordinate publication and release dates.

LICENSE AND PAYMENT:

All articles published in EMBO Molecular Medicine are fully open access: immediately and freely available to read, download and share.

EMBO Molecular Medicine charges an article processing charge (APC) to cover the publication costs. You, as the corresponding author for this manuscript, should have already received a quote with the article processing fee separately. Please let us know in case this quote has not been received.

Once your article is at Wiley for editorial production you will receive an email from Wiley's Author Services system, which will ask you to log in and will present you with the publication license form for completion. Within the same system the publication fee can be paid by credit card, an invoice, pro forma invoice or purchase order can be requested.

Payment of the publication charge and the signed Open Access Agreement form must be received before the article can be published online.

PROOFS

You will receive the proofs by e-mail approximately 2 weeks after all relevant files have been sent o our Production Office. Please return them within 48 hours and if there should be any problems, please contact the production office at embopressproduction@wiley.com.

Please inform us if there is likely to be any difficulty in reaching you at the above address at that time. Failure to meet our deadlines may result in a delay of publication.

All further communications concerning your paper proofs should quote reference number EMM-2023-17804-V4 and be directed to the production office at embopressproduction@wiley.com.

EMBO Press Author Checklist

Corresponding Author Name: Ignacio Melero and Maite Alvarez	
Journal Submitted to: EMBO Molecular Medicine	
Manuscript Number: EMM-2023-17804	

USEFUL LINKS FOR COMPLETING THIS FORM
The EMBO Journal - Author Guidelines
EMBO Reports - Author Guidelines
Molecular Systems Biology - Author Guidelines
EMBO Molecular Medicine - Author Guidelines

Reporting Checklist for Life Science Articles (updated January
This checklist is adapted from Materials Design Analysis Reporting (MDAR) Checklist for Authors. MDAR establishes a minimum set of requirements in transparent reporting in the life sciences (see Statement of Task: 10.31222/osf.io/9sm4x). Please follow the journal's guidelines in preparing your
Please note that a copy of this checklist will be published alongside your article.

Abridged guidelines for figures
1. Data
The data shown in figures should satisfy the following conditions:
- the data were obtained and processed according to the field's best practice and are presented to reflect the results of the experiments in an accurate and unbiased manner.
- ideally, figure panels should include only measurements that are directly comparable to each other and obtained with the same assay.
- plots include clearly labeled error bars for independent experiments and sample sizes. Unless justified, error bars should not be shown for technical
- if n<5, the individual data points from each experiment should be plotted. Any statistical test employed should be justified.
- Source Data should be included to report the data underlying figures according to the guidelines set out in the authorship guidelines on Data

2. Captions
Each figure caption should contain the following information, for each panel where they are relevant:
- a specification of the experimental system investigated (eg cell line, species name).
- the assay(s) and method(s) used to carry out the reported observations and measurements.
- an explicit mention of the biological and chemical entity(ies) that are being measured.
- an explicit mention of the biological and chemical entity(ies) that are altered/varied/perturbed in a controlled manner.
- the exact sample size (n) for each experimental group/condition, given as a number, not a range;
- a description of the sample collection allowing the reader to understand whether the samples represent technical or biological replicates (including how many animals, litters, cultures, etc.).
- a statement of how many times the experiment shown was independently replicated in the laboratory.
- definitions of statistical methods and measures:
  - common tests, such as t-test (please specify whether paired vs. unpaired), simple χ2 tests, Wilcoxon and Mann-Whitney tests, can be unambiguously identified by name only, but more complex techniques should be described in the methods section;
  - are tests one-sided or two-sided?
  - are there adjustments for multiple comparisons?
  - exact statistical test results, e.g., P values = x but not P values < x;
  - definition of 'center values' as median or average;
  - definition of error bars as s.d. or s.e.m.

Please complete ALL of the questions below.
Select "Not Applicable" only when the requested information is not relevant for your study.

Materials

Newly Created Materials	Information included in the manuscript?	In which section is the information available? (Reagents and Tools Table, Materials and Methods, Figures, Data Availability Section)
New materials and reagents need to be available; do any restrictions apply?	Not Applicable	

Antibodies	Information included in the manuscript?	In which section is the information available? (Reagents and Tools Table, Materials and Methods, Figures, Data Availability Section)
For **antibodies** provide the following information:<br>- Commercial antibodies: RRID (if possible) or supplier name, catalogue number and or/clone number<br>- Non-commercial: RRID or citation	Yes	Appendix Table S1

DNA and RNA sequences	Information included in the manuscript?	In which section is the information available? (Reagents and Tools Table, Materials and Methods, Figures, Data Availability Section)
**Short novel DNA or RNA including primers, probes**: provide the sequences.	Not Applicable	

Cell materials	Information included in the manuscript?	In which section is the information available? (Reagents and Tools Table, Materials and Methods, Figures, Data Availability Section)
**Cell lines:** Provide species information, strain. Provide accession number in repository **OR** supplier name, catalog number, clone number, and/**OR** RRID.	Yes	Material and Methods: Cell lines
**Primary cultures:** Provide species, strain, sex of origin, genetic modification status.	Not Applicable	
Report if the cell lines were recently **authenticated** (e.g., by STR profiling) and tested for mycoplasma contamination.	Yes	Material and Methods: Cell lines

Experimental animals	Information included in the manuscript?	In which section is the information available? (Reagents and Tools Table, Materials and Methods, Figures, Data Availability Section)
**Laboratory animals or Model organisms:** Provide species, strain, sex, age, genetic modification status. Provide accession number in repository **OR** supplier name, catalog number, clone number, **OR** RRID.	Yes	Material and Methods: Mice
**Animal observed in or captured from the field:** Provide species, sex, and age where possible.	Not Applicable	
Please detail **housing and husbandry conditions**.	Yes	Material and Methods: Mice

Plants and microbes	Information included in the manuscript?	In which section is the information available? (Reagents and Tools Table, Materials and Methods, Figures, Data Availability Section)
**Plants:** provide species and strain, ecotype and cultivar where relevant, unique accession number if available, and source (including location for collected wild specimens).	Not Applicable	
**Microbes:** provide species and strain, unique accession number if available, and source.	Not Applicable	

Human research participants	Information included in the manuscript?	In which section is the information available? (Reagents and Tools Table, Materials and Methods, Figures, Data Availability Section)
If collected and within the bounds of privacy constraints report on age, sex and gender or ethnicity for all study participants.	Yes	Material and Methods: Human materials

Core facilities	Information included in the manuscript?	In which section is the information available? (Reagents and Tools Table, Materials and Methods, Figures, Data Availability Section)
If your work benefited from core facilities, was their service mentioned in the acknowledgments section?	Yes	Acknowledgments

Design

Study protocol	Information included in the manuscript?	In which section is the information available? (Reagents and Tools Table, Materials and Methods, Figures, Data Availability Section)
If study protocol has been **pre-registered, provide DOI in the manuscript**. For clinical trials, provide the trial registration number **OR** cite DOI.	Not Applicable	
Report the **clinical trial registration number** (at ClinicalTrials.gov or equivalent), where applicable.	Not Applicable	

Laboratory protocol	Information included in the manuscript?	In which section is the information available? (Reagents and Tools Table, Materials and Methods, Figures, Data Availability Section)
Provide DOI OR other citation details if **external detailed step-by-step protocols** are available.	Not Applicable	

Experimental study design and statistics	Information included in the manuscript?	In which section is the information available? (Reagents and Tools Table, Materials and Methods, Figures, Data Availability Section)
Include a statement about **sample size** estimate even if no statistical methods were used.	Yes	Material and Methods: Mice
Were any steps taken to minimize the effects of subjective bias when allocating animals/samples to treatment (e.g. **randomization procedure**)? If yes, have they been described?	Yes	Material and Methods: Mice
Include a statement about **blinding** even if no blinding was done.	Yes	Material and Methods: Mice
Describe **inclusion/exclusion criteria** if samples or animals were excluded from the analysis. Were the criteria pre-established?<br><br>If sample or data points were omitted from analysis, report if this was due to attrition or intentional exclusion and provide justification.	Not Applicable	
For every figure, are **statistical tests** justified as appropriate? Do the data meet the assumptions of the tests (e.g., normal distribution)? Describe any methods used to assess it. Is there an estimate of variation within each group of data? Is the variance similar between the groups that are being statistically compared?	Yes	Material and Methods: Statistical Analysis

Sample definition and in-laboratory replication	Information included in the manuscript?	In which section is the information available? (Reagents and Tools Table, Materials and Methods, Figures, Data Availability Section)
In the figure legends: state number of times the experiment was **replicated** in laboratory.	Yes	Figure Legends for each figure
In the figure legends: define whether data describe **technical or biological replicates**.	Yes	Figure Legends for each figure

Ethics

Ethics	Information included in the manuscript?	In which section is the information available? (Reagents and Tools Table, Materials and Methods, Figures, Data Availability Section)
Studies involving **human participants**: State details of **authority granting ethics approval** (IRB or equivalent committee(s), provide reference number for approval.	Yes	Material and Methods: Human materials
Studies involving **human participants**: Include a statement confirming that **informed consent** was obtained from all subjects and that the experiments conformed to the principles set out in the WMA Declaration of Helsinki and the Department of Health and Human Services Belmont Report.	Yes	Material and Methods: Human materials
Studies involving **human participants:** For publication of **patient photos**, include a statement confirming that consent to publish was obtained.	Not Applicable	
Studies involving experimental **animals**: State details of **authority granting ethics approval** (IRB or equivalent committee(s), provide reference number for approval. Include a statement of compliance with ethical regulations.	Yes	Material and Methods: Mice
Studies involving **specimen and field samples:** State if relevant **permits** obtained, provide details of authority approving study; if none were required, explain why.	Not Applicable	

Dual Use Research of Concern (DURC)	Information included in the manuscript?	In which section is the information available? (Reagents and Tools Table, Materials and Methods, Figures, Data Availability Section)
Could your study fall under dual use research restrictions? Please check biosecurity documents and list of **select agents and toxins** (CDC): https://www.selectagents.gov/sat/list.htm	Not Applicable	
If you used a select agent, is the security level of the lab appropriate and reported in the manuscript?	Not Applicable	
If a study is subject to dual use research of concern regulations, is the name of the **authority granting approval and reference number** for the regulatory approval provided in the manuscript?	Not Applicable	

Reporting

The MDAR framework recommends adoption of discipline-specific guidelines, established and endorsed through community initiatives. Journals have their own policy about requiring specific guidelines and recommendations to complement MDAR.

Adherence to community standards	Information included in the manuscript?	In which section is the information available? (Reagents and Tools Table, Materials and Methods, Figures, Data Availability Section)
State if relevant guidelines or checklists (e.g., **ICMJE, MIBBI, ARRIVE, PRISMA**) have been followed or provided.	Not Applicable	
For **tumor marker prognostic studies,** we recommend that you follow the **REMARK** reporting guidelines (see link list at top right). See author guidelines, under 'Reporting Guidelines'. Please confirm you have followed these guidelines.	Not Applicable	
For **phase II and III randomized controlled trials**, please refer to the **CONSORT** flow diagram (see link list at top right) and submit the CONSORT checklist (see link list at top right) with your submission. See author guidelines, under 'Reporting Guidelines'. Please confirm you have submitted this list.	Not Applicable	

Data Availability

Data availability	Information included in the manuscript?	In which section is the information available? (Reagents and Tools Table, Materials and Methods, Figures, Data Availability Section)
Have **primary datasets** been deposited according to the journal's guidelines (see 'Data Deposition' section) and the respective accession numbers provided in the Data Availability Section?	Not Applicable	
Were **human clinical and genomic datasets** deposited in a public access-controlled repository in accordance to ethical obligations to the patients and to the applicable consent agreement?	Not Applicable	
Are **computational models** that are central and integral to a study available without restrictions in a machine-readable form? Were the relevant accession numbers or links provided?	Not Applicable	
If publicly available data were reused, provide the respective **data citations in the reference list**.	Not Applicable	
